# Path integration deficits are associated with phosphorylated tau accumulation in the entorhinal cortex

**DOI:** 10.1093/braincomms/fcad359

**Published:** 2024-02-12

**Authors:** Riki Koike, Yoshiyuki Soeda, Atsushi Kasai, Yusuke Fujioka, Shinsuke Ishigaki, Akihiro Yamanaka, Yuta Takaichi, James K Chambers, Kazuyuki Uchida, Hirohisa Watanabe, Akihiko Takashima

**Affiliations:** Laboratory for Alzheimer’s Disease, Department of Life Science, Faculty of Science, Gakushuin University, Toshima-ku, Tokyo 171-8588, Japan; Laboratory for Alzheimer’s Disease, Department of Life Science, Faculty of Science, Gakushuin University, Toshima-ku, Tokyo 171-8588, Japan; Deapartment of Research and Development, MIG (Medical Innovation Group) Inc, Shibuya, Tokyo 150-0031, Japan; Molecular Neuroscience Research Center, Shiga University of Medical Science, Otsu, Shiga 520-2192, Japan; Molecular Neuroscience Research Center, Shiga University of Medical Science, Otsu, Shiga 520-2192, Japan; Department of Neuroscience II, Research Institute of Environmental Medicine, Nagoya University, Nagoya, Aichi 464-8601, Japan; Laboratory of Veterinary Pathology, Graduate School of Agricultural and Life Sciences, The University of Tokyo, Bunkyo-ku, Tokyo 113-8657, Japan; Laboratory of Veterinary Pathology, Graduate School of Agricultural and Life Sciences, The University of Tokyo, Bunkyo-ku, Tokyo 113-8657, Japan; Laboratory of Veterinary Pathology, Graduate School of Agricultural and Life Sciences, The University of Tokyo, Bunkyo-ku, Tokyo 113-8657, Japan; Department of Neurology, Fujita Health University, Toyoake, Aichi 470-1192, Japan; Laboratory for Alzheimer’s Disease, Department of Life Science, Faculty of Science, Gakushuin University, Toshima-ku, Tokyo 171-8588, Japan

**Keywords:** neurofibrillary tangle (NFT), entorhinal cortex, path integration, 3D virtual reality task

## Abstract

Alzheimer’s disease is a devastating disease that is accompanied by dementia, and its incidence increases with age. However, no interventions have exhibited clear therapeutic effects. We aimed to develop and characterize behavioural tasks that allow the earlier identification of signs preceding dementia that would facilitate the development of preventative and therapeutic interventions for Alzheimer’s disease. To this end, we developed a 3D virtual reality task sensitive to the activity of grid cells in the entorhinal cortex, which is the region that first exhibits neurofibrillary tangles in Alzheimer’s disease. We investigated path integration (assessed by error distance) in a spatial navigation task sensitive to grid cells in the entorhinal cortex in 177 volunteers, aged 20–89 years, who did not have self-reported dementia. While place memory was intact even in old age, path integration deteriorated with increasing age. To investigate the relationship between neurofibrillary tangles in the entorhinal cortex and path integration deficit, we examined a mouse model of tauopathy (P301S mutant tau-overexpressing mice; PS19 mice). At 6 months of age, PS19 mice showed a significant accumulation of phosphorylated tau only in the entorhinal cortex, associated with impaired path integration without impairments in spatial cognition. These data are consistent with the idea that path integration deficit is caused by the accumulation of phosphorylated tau in the entorhinal cortex. This method may allow the early identification of individuals likely to develop Alzheimer’s disease.

## Teaser

Koike et.al. developed a 3D virtual reality task (path integration) sensitive to the grid cell activity in the entorhinal cortex, which is the region that first exhibits neurofibrillary tangles in Alzheimer’s disease. They found that path integration deficit is related to an accumulation of phosphorylated tau in the entorhinal cortex.

## Graphical Abstract

**Graphical Abstract:**
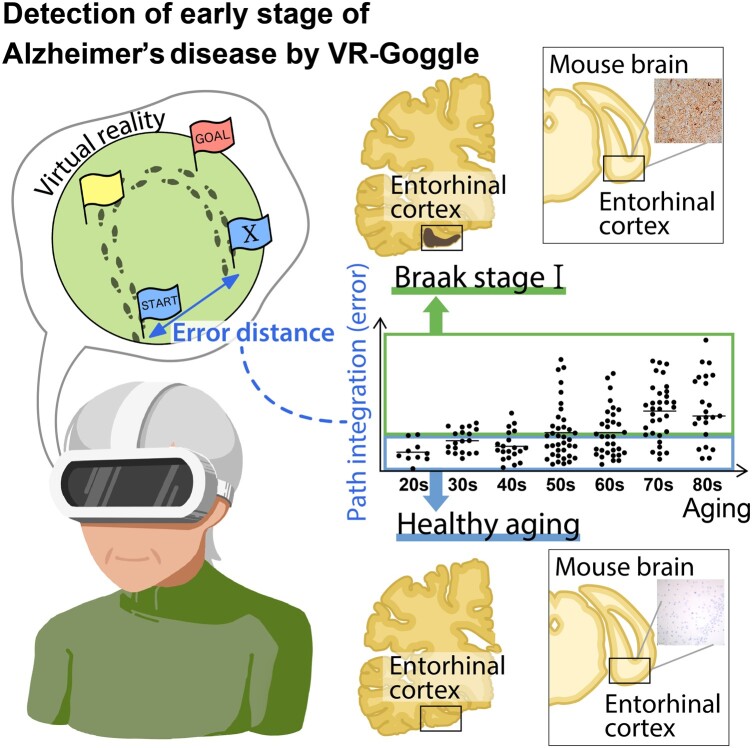

See Almeida (https://doi.org/10.1093/braincomms/fcae014) for a scientific commentary on this article.

## Introduction

Alzheimer’s disease is a devastating disease that occurs in older persons. Its main symptom is dementia, and its neuropathological features include β-amyloid accumulation, neurofibrillary tangles (NFTs) composed of phosphorylated tau and brain atrophy. Each of these pathological changes is also observed during normal brain ageing. With the growth in the ageing population worldwide, the incidence of Alzheimer’s disease is likely to grow. Developing preventative (or ameliorating) therapeutics against Alzheimer’s disease has so far been limited because early detection of the underlying pathology has proven difficult.^1,2^

Age-related development of NFTs first emerges in the entorhinal cortex (EC). Over time, NFTs spread from the EC (Stages I and II) to the limbic cortex (Stages III and IV) and neocortex (Stages V and VI). Although NFTs in the EC are not accompanied by signs of dementia, NFTs in the limbic areas and neocortex are usually associated with mild cognitive impairment (MCI) or even overt dementia.^3,4^ Thus, NFT stages reflect clinical brain ageing, and pathological changes in the EC (i.e. the accumulation of NFTs) are the earliest pathological indicator of the potential conversion from a pre-clinical state to clinical deterioration.^5-7^ Because NFTs accumulate in the EC before memory problems,^7,8^ the early identification of NFTs in the EC may be useful for the prevention of Alzheimer’s disease. Recently, it has been reported that increased plasma phosphorylated tau concentration correlates with NFT density and precedes β-amyloid accumulation,^9-11^ and measurement of plasma phosphorylated tau concentration is expected to be a diagnostic marker for early Alzheimer’s disease.^12^ Notably, however, these measurements do not necessarily reflect the extent of NFT in the EC.

Navigation is the process of determining and maintaining a route between different points on a spatial map; the behaviour relies on brain regions implicated in Alzheimer’s disease (e.g. EC, hippocampus, parietal cortex, precuneus, retrosplenial cortex).^13^ The EC contains grid cells that provide the microstructure of a spatial map, generate specific periodic representations of self-location^14^ and contribute to path integration performance^15-17^; manipulations that impact grid cell activity also impair path integration.^18^ In light of neuropathological findings showing that Alzheimer’s disease pathology is initiated in the EC, the purpose of this work was to develop a cognitive (navigation) test that selectively engages grid cells in the EC ^14^ and would therefore be sensitive to disruption of the EC, a phenomenon hypothesized to occur Alzheimer’s disease.^13^

The rationale for studying navigation deficits was based on the fact that (i) carriers of the apolipoprotein E ε4 allele exhibit reduced path integration abilities and reduced grid cell-like activity decades before disease onset,^17,19,20^ (ii) the decline of path integration predicts the risk of Alzheimer’s disease in midlife^21^ and (iii) patients with diagnosed MCI exhibit significant impairments in path integration that correlate with EC volume.^16^ While the aforementioned observations suggest that declining path integration may be predictive of Alzheimer’s disease, a causal relationship between pathological changes in the EC and deficits in path integration has not been demonstrated so far.

To evaluate these relationships and the underlying mechanisms, path integration in volunteers aged 20–89 years was investigated. Further, we used a mouse model of tauopathy to examine whether deficits in path integration correlate with the extent of NFTs in the EC. Our findings that deficits in path integration in mice increase with accumulation of phosphorylated tau in the EC only, i.e. before spreading to other cortico-limbic structures, suggest that impairments in path integration in humans may predict the expected increases in NFT in the EC.

## Materials and methods

### Participants

We enrolled participants who agreed to the research procedures by signing an informed consent form and could travel by themselves to the testing site. The enrolment and the test were conducted from December 2019 to December 2020. All participants were Japanese volunteers who self-reported that they were free of dementia. Exclusion criteria and group sizes were decided on the basis of previous work.^15^ Subjects reporting a neurological or psychiatric disorder were excluded from the study. All participants had normal or corrected-to-normal vision. The numbers of female and male participants in each of the age groups are shown in Table 1. Human autopsy brain data are taken from Braak and Braak’s paper.^22^ The study was performed in line with the regulations outlined in the Declaration of Helsinki (WMA, 2013) and approved by the Research Ethics Committee of Gakushuin University.

**Table 1:** Human subject ages and numbers

	Spatial cognition				Path integration		
	Age (years)	Female	Male		Age (years)	Female	Male
		*n* (%)	*n* (%)			*n* (%)	*n* (%)
20s	23.2 ± 3.7	5 (50.0)	5 (50.0)		23.2 ± 3.7	5 (50.0)	5 (50.0)
30s	34.7 ± 3.1	10 (52.6)	9 (47.4)		34.7 ± 3.1	10 (52.6)	9 (47.4)
40s	44.7 ± 2.9	10 (50.0)	10 (50.0)		44.6 ± 2.9	9 (47.4)	10 (52.6)
50s	54.3 ± 2.6	21 (56.8)	16 (43.2)		54.4 ± 2.6	20 (55.6)	16 (44.4)
60s	64.1 ± 3.0	18 (54.5)	15 (45.5)		64.0 ± 3.0	19 (55.9)	15 (44.1)
70s	73.5 ± 2.9	13 (39.4)	20 (60.6)		73.5 ± 2.9	11 (35.5)	20 (64.5)
80s	82.0 ± 2.1	5 (21.7)	18 (78.3)		82.1 ± 2.1	5 (22.7)	17 (77.3)

Age is expressed as mean ± SD.

## Virtual reality test

The virtual reality (VR) space consists of a 20 virtual meters (vm) diameter virtual arena surrounded by 3 vm high walls. Subjects donned 3D VR goggles and operated their movements with a joystick (Meta Quest 2). At first, they were allowed to move freely through the virtual arena with obstacles to familiarize themselves with the setup. Forward and backward movements were controlled with the joystick, while left and right movements were controlled by rotating their body while seated on a swivel chair. Volunteers with visual problems or those who could not see and use the joystick properly were excluded. Subjects who showed VR sickness were excluded from the analysis (data from 2 subjects in the path integration test and data from 6 subjects in the spatial cognition test were excluded). The software and VR goggles have been described previously ^16^ and were provided by MIG Inc. (Tokyo, Japan; https://www.medicalig.com) and used. The assessment was divided into two parts: a path integration test, followed by a break of 5–10 min, and a spatial cognition test. A total of 177 subjects provided informed consent for the tests and signed the informed consent form. However, due to exclusions caused by errors in the testing procedure or a subject’s inability to complete the test, we obtained data from 175 subjects on the path integration test and 171 subjects on the spatial cognition test (Table 1). No differences in path integration were found between men and women.

### Spatial cognition

**Figure 1:**
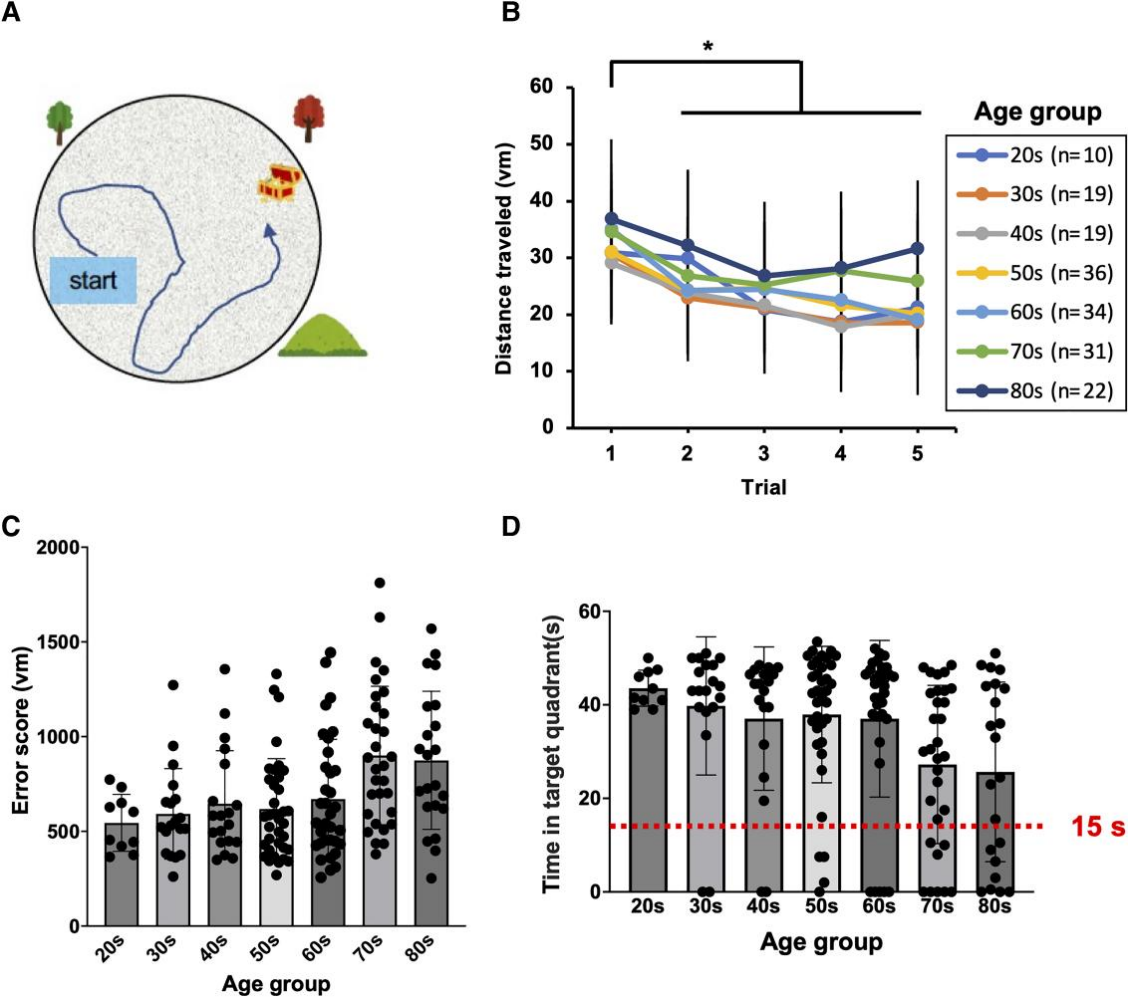
**Spatial learning and memory abilities in human subjects.** (**A**) Spatial learning and memory abilities in human subjects (*n* = 171) were evaluated (see Materials and Methods for details). Participants searched for hidden treasure chests in an arena. (**B**) The distance travelled from the start to the hidden goal was measured as spatial learning. The results are expressed as the mean ± SD. A significant difference (*P < 0.05) was observed in all age groups between the distance in Trial 1 and that in Trials 2, 3, 4 and 5 using repeated-measures ANOVA and Dunnett's multiple comparisons test. The probe trial examined spatial memory by removing the hidden goals; this 60-s trial was carried out after the spatial learning shown in **B**. Spatial memory was assessed in terms of the error score, the integral distance between the hidden goal and participant's position (**C**, see Supplemental Table 2) and time spent in the target quadrant (**D**, see Supplemental Table 3). Subjects that spent 15 s (a dashed line) or less in the target quadrant, which means they were at the target quadrant for less than a quarter of the trial time, were considered to lack a spatial memory of the location. The results are expressed as the mean ± SD, and Kruskal–Wallis and Dunn's multiple comparisons tests were applied (**C** and **D**). Detailed statistical analyses are given in Supplemental Tables 1–3.

The spatial cognition test was conducted in a 3D VR space using VR goggles (Oculus Quest, Menlo Park, CA, USA) provided by MIG Inc. Subjects had 1 min to find a hidden treasure chest in an arena with a diameter of 20 vm (Fig. 1A). If the subjects did not find the treasure chest, its location was revealed, and the subjects virtually moved to that location for further exploration for 30 s. This process was repeated five times, so that subjects learned the location of the treasure chest. Finally, in the probe trial, the treasure chest was removed, and the subject was allowed 1 min to search for the treasure chest. The individual’s starting point in the arena was different on each trial. The distance to reach the goal in each trial was measured and used to assess learning. In the probe trial, the arena was divided into four quadrants, and the percentage of time spent in the target quadrant (the theoretical location of the goal) out of the total search time was used as an index of spatial memory. Spatial memory was also assessed according to the error score, which was the sum of the distance from the goal measured every 0.5 s during the search.

### Path integration

**Figure 2:**
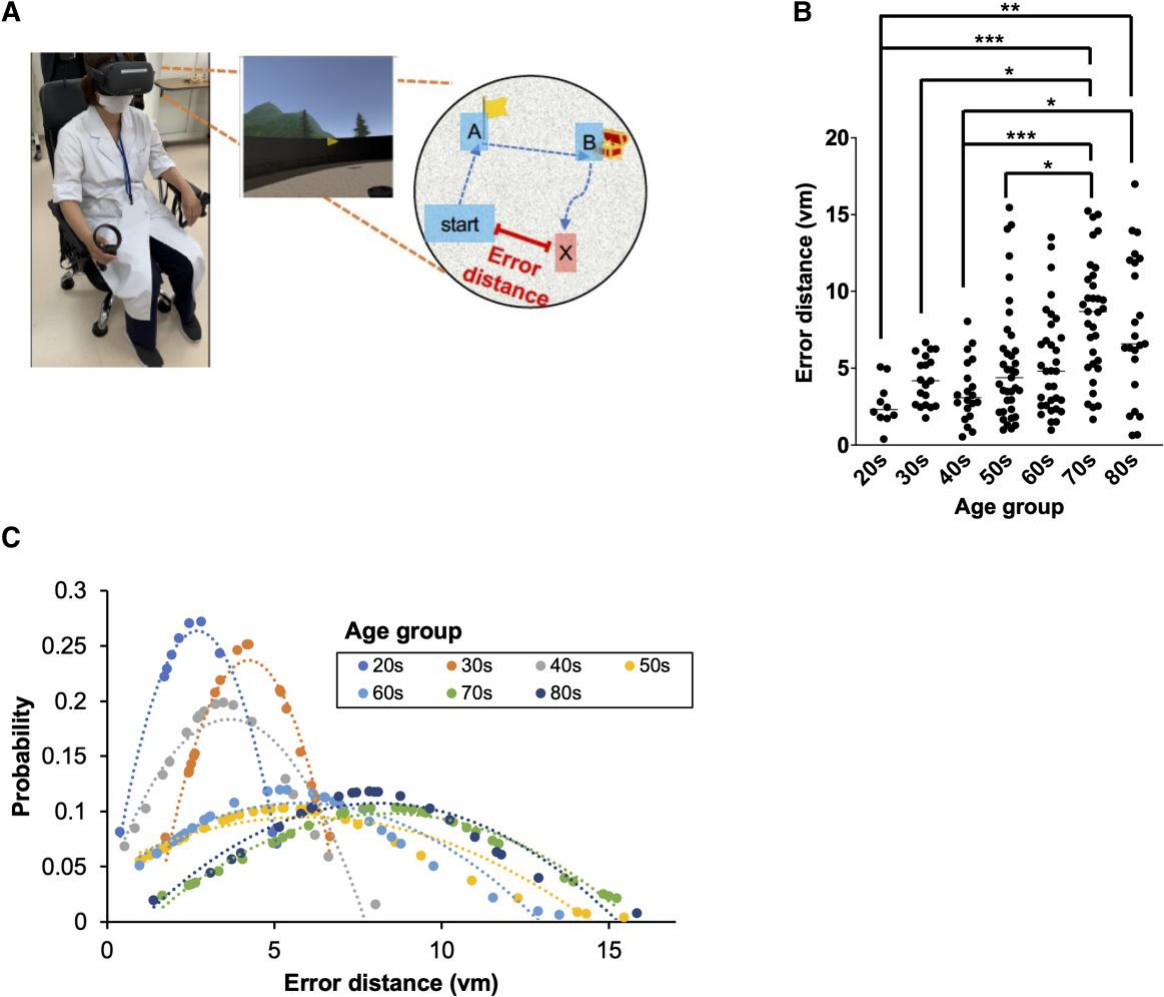
**Path integration in human subjects.** (**A**) The path integration of human subjects (*n* = 175) was evaluated. Path integration was measured by having the participant, wearing VR goggles, go to Location A (indicated by a yellow flag), then to Location B (indicated by a red flag) and finally return to the starting point. The error distance was considered the error distance between the participant's final location (x) and the actual starting point. (**B**) The error distance for each individual was calculated as the average of three trials, and these results are expressed as the mean; dots represent individual mean values. *P*-values were determined using Kruskal–Wallis tests and Dunn's multiple comparisons tests. *P < 0.05, **P < 0.01 and ***P < 0.001. (**C**) The probability of each error distance value plotted for all age groups. According to an F test, significant dispersion was observed in the subjects in their 50, 60, 70 and 80 s compared with the subjects in their 20, 30 and 40 s (see Supplemental Table 5). Detailed data analyses are given Supplemental Tables 4 and 5.

Path integration performance was measured by having the subjects while wearing the VR goggles, go to an indicated Location A (yellow flag), then to a different indicated Location B (red flag) and finally return to the starting point (Fig. 2A). The return time is 1 min, and the position after 1 min is the final position as considered the starting position by the subject. The distance between the subject’s final position and the actual starting point (the error distance) was measured. The subjects took this test three times, and the average error distance was used as an index of path integration. All three trials started from different locations and returned from different locations, but the distance from the location of the last flag to the starting point was held constant.

### Animals

Wild-type (WT; C57BL/6J) mice were purchased from Japan SLC (Hamamatsu, Japan). PS19 mice ^23^ that expressed T34 tau (1N4R) with an FTDP17 mutation (P301S) driven by the *PRNP* promoter were obtained from the Jackson Laboratory and, through successive generations, maintained the C57BL/6J genetic background.

All animals were single-housed and kept on a 12 h light/12 h dark cycle, with free access to food and water; behavioural tests were conducted on female and male mice. Injured or weakened mice due to fighting or other reasons were excluded from the experiment. All animal experiments were approved by the Gakushuin University Animal Experimentation Committee. The numbers and sex distribution of animals in each experiment are shown in Supplemental Tables 6 and 7.

## Designer receptors exclusively activated by designer drug system

To investigate the functional properties of the neurons in the medial EC (MEC) underlying path integration, we used a designer receptors exclusively activated by designer drug (DREADD) system that employed adeno-associated viruses (AAVs) expressing hM4Di coupled with subsequent CNO (R&D Systems, Minneapolis, MN, USA) administration. We injected 1.0 μl of AAV-hSyn-mCherry-hM4Di virus (3.3 × 10^12^ copies/ml) into the bilateral MEC regions (L: +/−3.2, A: −4.48, H: −4.50) of 8-week-old male mice (C57BL/6J) at a flow rate of 0.25 μl/min. The injection cannula was maintained in place for 3 min post-injection. Animals were handled daily for 1 week before the injections and were also habituated to dummy injections. One hour before testing in an L-maze, mice received vehicle (10% DMSO) or CNO [1.0 μg/g of BW, 0.1 μg/μl in 10% DMSO (Nacalai Tesque, Kyoto, Japan) saline mixture]. A cross-over design was used 1 day later (switch between CNO-injected and vehicle-injected groups), which used different field and goal positions in the L-maze. Two weeks later, the same mice received vehicle or CNO (as above) and were exposed to the L-maze test. Mice were sacrificed by decapitation 1 h after the maze test when their brains were fixed by perfusion with 4% PFA before being processed for c-Fos staining (see below). Images were obtained using an all-in-one fluorescence microscope (BZ-X700, KEYENCE, Osaka, Japan) equipped with a Plan Apochromat 40× objective (NA0.95, BZ-PA40, KEYENCE). Seven images including the EC were randomly selected from each group, and the density of c-Fos–positive cells per mm^2^ in the MEC (Supplemental Fig. 2) was semi-automatically quantified using a hybrid cell count application (BZ-H4C, KEYENCE) using BZ-X Analyzer software (BZ-H4A, KEYENCE).

### Immunohistochemistry

Brains were fixed by immersion in formalin before embedding in paraffin wax and coronal sectioning (4 or 8 μm), and consecutive sections were used for immunohistochemistry. Briefly, endogenous peroxidase activity was blocked with 1% hydrogen peroxide in methanol for 5 min, and non-specific antibody binding was blocked using 8% skim milk in Tris-buffered saline. sections were subsequently incubated with primary antibodies—mouse anti-hp-tau (Ser202/Thr205; clone AT8, 1:500, Thermo Scientific, Rockford, IL, USA) or anti-c-Fos [Anti-c-Fos antibody (sc166940) Mouse, 1/100]—overnight at 4°C. Immunolabelled antigens were then visualized using the Dako EnVision+ System (Dako, Glostrup, Denmark) with 0.02% 3′3-diaminobenzidine plus 0.01% hydrogen peroxide as a chromogen or FITC-labelled secondary antibody.

### L-maze test

The L-maze test was performed in a circular field (1 m in diameter), which was the same field as that used for the Barnes maze test, with a movable cylinder in the centre and 12 holes (4 cm in diameter) along the edge. An escape box (13 × 17 × 7 cm) was placed under each hole. The field was illuminated from above (∼1000 lux), and a square start box (13 × 13 × 22.5 cm) with only one exit was placed in the centre of the field. The apparatus was surrounded by a curtain to occlude visual cues, except for when a visual cue was presented. In the latter case, a plastic box was placed at a height of 40 cm outside the maze.

Experiments in the L-maze were commenced following 1 week of habituation to handling and exposure to the test field. In the conditioning phase, an L-shaped maze, which consisted of a long walkway (33.5 × 6 × 22.5 cm) and a short walkway (25.5 × 6 × 22.5 cm) at a right angle, was connected to the start box. The mouse travelled from the start box to the escape box, located at the end of the L-shaped maze. The behaviour of the mice was recorded with a CCD camera, and the position coordinate data (*x*, *y*) were acquired by TimeBCM (O’HARA & CO., LTD., Tokyo, Japan) at 10 frames/s.

Path integration performance was assessed after slight modifications of previously described protocols.^24^ The behavioural paradigm consisted of three phases (the training, conditioning and test phases). Habituation for the test field and conditioning/tests were performed using different hemispheres of the Barnes maze. In the conditioning phase, the L-shaped maze was connected to the start box, and mice travelled to the escape box, which was in a fixed position. After reaching the escape box, mice remained there for 1 min and were then returned to the home cage. The conditioning phase was repeated five times to allow acclimation to the spatial arrangement of the start box and the location of escape without a visual cue. These trial and test sequences were interspersed by an interval of 15 min. In the test phase after conditioning, the L-shaped maze was removed, and the mice were allowed to freely travel from the start box to the escape box. The session was terminated at the mice reached the hole or 3 min after starting. Path integration performance in the L-maze test was expressed as angular error, which was the average angle between the path of the mouse for the first 20 cm and the angle of the goal. The angle at which the mouse would reach the goal after travelling in a straight line from the starting position was 0°, and the angular error from the starting point was calculated for each mouse until it reached 20 cm from the starting point, using the point where the mouse left the start box as the starting point.

### Barnes maze

Hippocampal-dependent spatial learning and memory were assessed in the Barnes maze.^25^ The goal was pseudorandomly chosen from among the 12 holes in the field that led to a dark escape box, which is preferred by mice. The field was brightly lit, and the mice were placed in the centre of the field and allowed to search for the dark escape box for 3 min. The mice that failed to find the escape box were led to its location. In the learning phase (Days 1–3), mice explored the field three times a day with intertrial intervals of 1 h. The arena was cleaned with 70% ethanol between trials. On the fourth day (probe trial), the escape box was removed, and mice were allowed to search for 3 min. For the behavioural analysis, we compared the distance travelled to enter the escape box (total distance travelled) in the learning trials and the duration that the mice spent in each quadrant of the field (percentage of time spent in the target quadrant), as well as the total distance from the goal (error score) for each time-series position, coordinate in the probe trial.

### Statistical analysis

All statistical (*post hoc*) analyses were conducted using GraphPad Prism 6 (GraphPad, La Jolla, CA, USA). Data from human subjects in the probe trial and the path integration test were analysed by non-parametric Kruskal–Wallis and Dunn’s multiple comparisons tests. Dispersion of error distance was analysed by F test. Repeated data from the learning trials were analysed by repeated-measures ANOVA and Dunnett’s multiple comparisons test. The probability distribution of error distance for each age group was calculated by the NORMDIST function using Microsoft Excel®. Mouse behavioural data were analysed by unpaired and paired *t*-test, a two-way ANOVA, Tukey’s multiple comparisons test and Bonferroni’s multiple comparisons test. Analyses of variance (F test) were performed prior to *t*-tests. Quantitative data from immunohistochemistry in PS19 mice were analysed by one-way ANOVA and Dunnett’s multiple comparisons test. A *P*-value <0.05 was considered statistically significant. Detailed statistical analyses are shown in Supplemental Tables 1–5 and 8–15.

## Results

### Ageing impairs path integration, but not spatial memory in a VR task

#### Maintenance of spatial memory across ages in humans

To investigate age-dependent changes in brain function, we studied path integration and spatial memory in volunteers ranging from 20 to 89 years old. All volunteers self-reported that they were free of dementia, and their spatial learning and spatial memory were assessed. We could only obtain results from 171 of the 177 participants (the residual displayed VR sickness). Spatial memory was assessed in these human volunteers using a task similar to the Morris water maze test in rats;^26^ subjects were given five learning trials in which they explored a specific location in a virtual 3D space using VR goggles (Fig. 1A), and spatial memory was then assessed with a probe trial. All age groups showed a similar travelling distance in the learning curve (Fig. 1B). In the probe trial, the error score, which is the total distance from the goal during the trial, tended to increase (Fig. 1C) with ageing, and the time spent in the target quadrant decreased with increasing age (Fig. 1D). However, even the oldest age group (subjects in their 80 s) spent at least 15 s in the target quadrant (Fig. 1D), suggesting that most subjects, including older subjects, maintained their spatial memory, even though it tended to decrease with ageing.

#### Impaired spatial navigation with increasing age in humans

Next, we investigated path integration in the same participants of varying ages who demonstrated the maintenance of spatial learning and memory. Results from 175 (out of 177) participants were obtained; 2 of the participants showed VR sickness. Path integration was measured by having the participant go to a specific Location A (yellow flag) in the virtual scene, then to a different Location B (red flag) and then return to the starting point (Fig. 2A). The distance between the subject’s final location and the actual point (the error distance) was used as an index of path integration performance. Figure 2B shows the error distances in the eight age groups, from subjects in their 20 s to those in their 80 s. The average error distance in each age group remained relatively constant through subjects in their 40 s but increased beginning from subjects in their 50 s to those in their 80 s. Compared with the average error distance for the 20 s age group, a significant increase in error distance was observed for the 70 and 80 s age groups. When the probability distributions were calculated for each age group (Fig. 2C), the error distances fell near the mean for subjects in their 20–40 s, while the error distances for those in their 50–80 s were much further from the mean. The variance in the probability distributions of error distances was similar in the 20–40 s age groups but increased significantly in the 50–80 s age groups (F test, *P* < 0.05). After the age of 50, individual error distances in the path integration test were substantially more variable, and the mean value increased, suggesting that the proportion of people with impaired EC-related navigation increases with age.

NFTs are rarely found in subjects in their 20 s^22^; and interestingly, the variance of error distance in this group in our study was small; accordingly, we used 5 vm (maximum error distance, confidence >95%) as a threshold to calculate the percentage of people with increased error distances in each age group. Accordingly, we observed that the proportion of individuals with an error distance in excess of increased with age, correlating with the expected proportion of individuals expected to have NFTs in the EC (Supplemental Fig. 1). This would imply that subjects with error distances >5 vm most likely display NFTs in the EC, an event that occurs early in the onset of Alzheimer’s disease.

### Accumulation of phosphorylated tau in the EC impairs path integration in mice

To investigate whether path integration performance is accompanied by NFT formation in the EC, we developed a behavioural paradigm to evaluate path integration in mice, using the so-called L-maze. We investigated the effects of suppressed EC activity, overexpression of mutant tau (in PS19 mice that overexpress tau in a manner that results in NFT formation) and distribution of tau pathology on path integration in this behavioural paradigm.

#### Suppression of EC activity with DREADDs and impaired path integration

**Figure 3:**
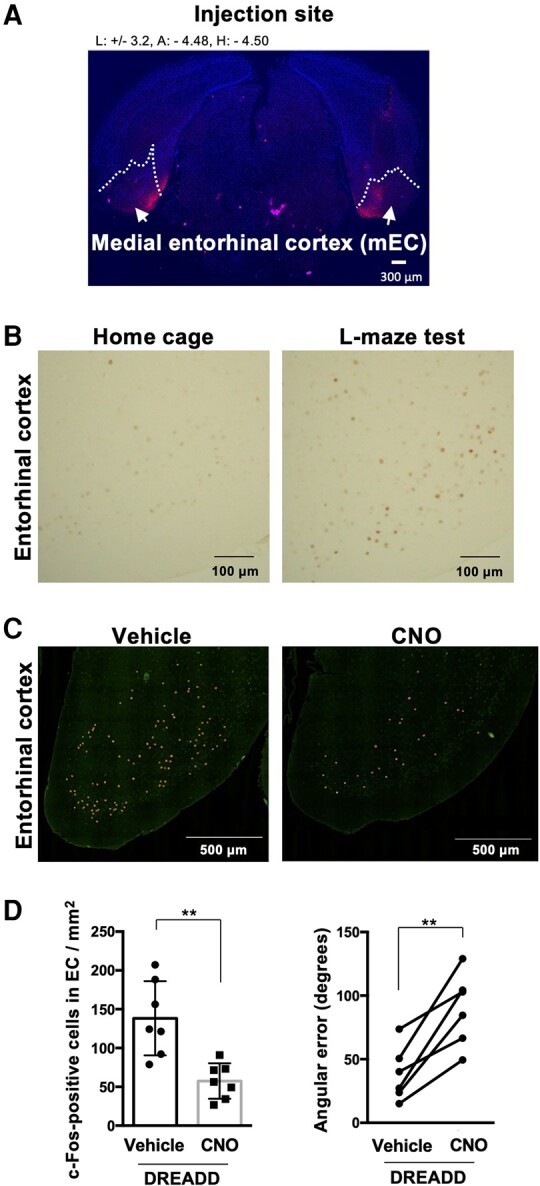
**Inhibition of neural activity in the EC impairs path integration in the L-maze test in mice.** (**A**) WT mice received a bilateral injection of AAV-hSyn-hM4Di-mCherry virus in the EC (L: +/− 3.2, A: −4.48, H: −4.50; dashed lines). The paraffin sections from the mice were immunostained with mCherry antibody (red) and counterstained with DAPI (blue). The area enclosed in dotted white line is the MEC. (**B**) c-Fos immunostaining of WT mouse EC staying homecage (left panel) and that of 1 hr after L-maze test (right panel). One hour before the L-maze test, the mice were intra-peritoneally injected with either CNO (1.0 μg/g of body weight; CNO, *n* = 3) or the same injection volume of 10% DMSO (10 μl/g of body weight; vehicle, *n* = 3), and an L-maze test was performed to examine the number of c-Fos-positive neurons (**C** and **D** left panel: CNO, *n* = 7; vehicle, *n* = 7; seven images including the EC were shown in Supplemental Fig. 2) and path integration (**D** right panel: *n* = 6), measured as the angular error. The effects of each injection were obtained from a cross-over experiment. Individual results after each indicated treatment are expressed as angular errors. *P*-values were determined using unpaired (**D** left) and paired (**D** right) *t*-tests. **P < 0.01. Detailed statistical analyses are given in Supplemental Tables 8 and 9.

First, we tested whether L-maze performance reflected neuronal activity in the EC. To inhibit neural activity in the EC in WT mice, we used DREADD-based chemogenetic tools. For this, AAV-hSyn-hM4Di-mCherry was bilaterally injected into the EC of 8-week-old WT mice (Fig. 3A). Following the L-maze test, the number of c-Fos–positive neurons in the EC was significantly increased (Fig. 3B), indicating L-maze test activated neurons in EC.

Blockade of neural activity in the EC by intraperitoneal administration of clozapine *N*-oxide (CNO; Fig. 3C and D left) impaired path integration in the L-maze in DREADD-treated mice versus vehicle-injected mice (Fig. 3D right). Path integration was not altered by CNO treatment in non–DREADD-expressing mice (Supplemental Fig. 3). These results demonstrate that path integration depends on neural activity in the EC.

#### Tau-overexpressing mice display impaired path integration

Phosphorylated tau levels begin to increase in the EC of 3-month-old and 6-month-old PS19 (tau-overexpressing mice). We, therefore, enquired how these pathological changes might impact path integration, an EC-dependent and hippocampus-dependent behaviour, in this mouse line.

**Figure 4:**
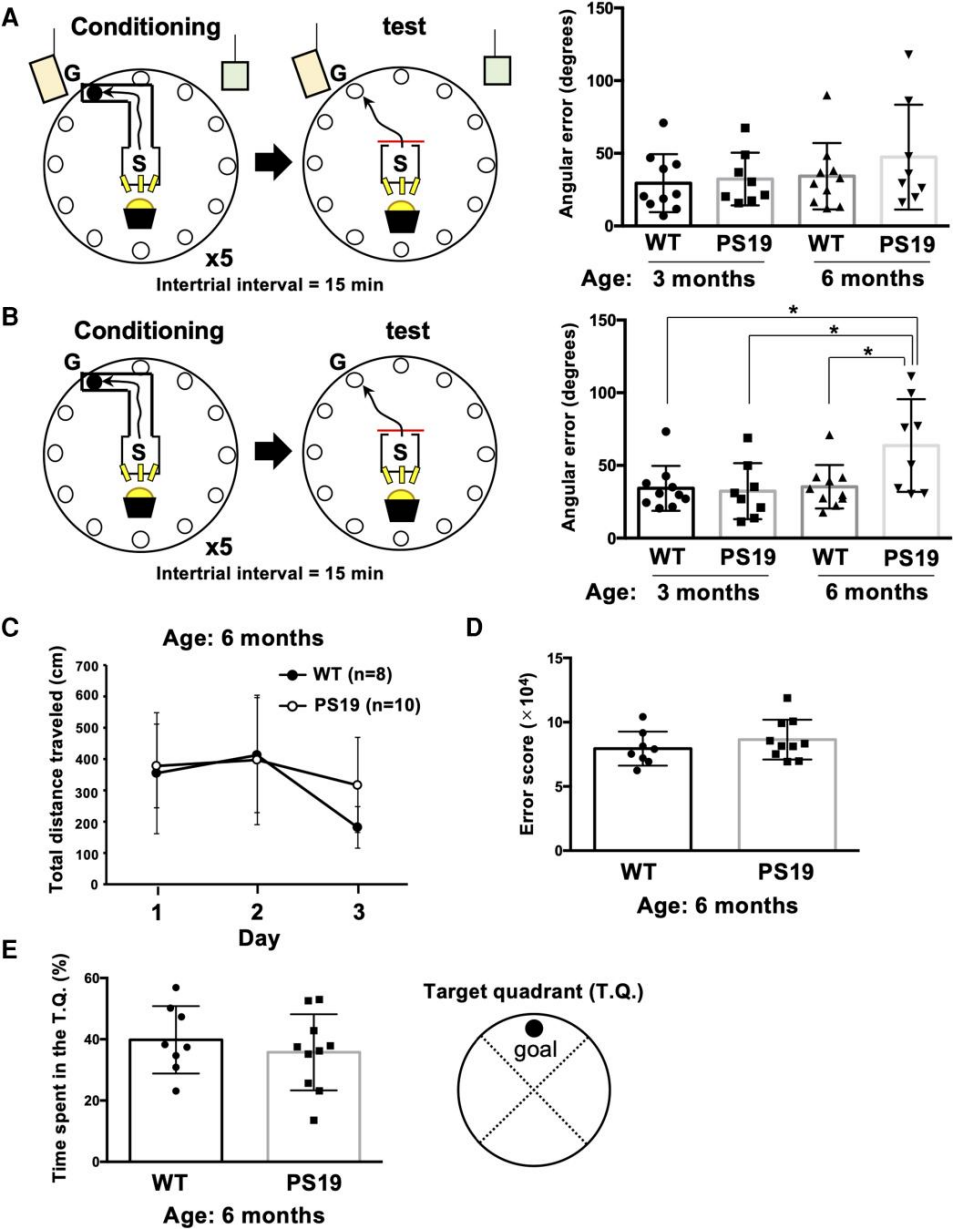
**PS19 mice exhibit impaired path integration but no change in spatial cognition at 6 months of age.** The path integration of PS19 mice was investigated in the L-maze test. (**A**) Under visual cue existence condition, WT (*n* = 10) and PS19 (*n* = 8) at 3 months of age and WT (*n* = 10) and PS19 (*n* = 8) at 6 months of age showed a similar angular error. (**B**) Without visual cue, the angular error of PS19 mice (*n* = 8) at 6 months of age was significantly higher than that of WT (*n* = 10, *P < 0.05) and PS19 (*n* = 8; *P < 0.05) at 3 months of age and WT (*n* = 10; *P < 0.05) at 6 months of age, indicating that PS19 mice at 6 months of age were impaired path integration. (**C**) In the Barnes maze, which assesses hippocampal-dependent spatial learning and memory, 6-month-old WT (*n* = 8) and PS19 (*n* = 10) mice showed no significant difference in the total distance travelled to the goal, but a significant effect of day was observed [two-way ANOVA; interaction, F(2,32) = 1.105, *P* = 0.3436; genotype, F(1,16) = 0.9802, *P* = 0.3369; day, F(2,32) = 4.735, *P < 0.05]. (**D** and **E**) In the Barnes maze probe trial, there was no significant difference in the error score (*P* = 0.3265) (**D**) or percentage of time spent in the target quadrant (**E**) between WT and PS19 mice (*P* = 0.4772). The results are expressed as the mean ± SD. *P*-values were determined using two-way ANOVA and Tukey's multiple comparisons tests (**A** and **B**, see Supplemental Tables 10 and 11), a two-way ANOVA (**C**, see Supplemental Table 12) and unpaired Student’s *t*-tests (**D and E**, see Supplemental Tables 13 and 14). *P < 0.05, **P < 0.01. Detailed data analyses are provided in Supplemental Tables 10–14. Representative paths of WT and PS19 mice in the L-maze test video are presented in Supplemental Video 1 (6-month-old WT) and Supplemental Video 2 (6-month-old PS19).

In the presence of a visual cue in the L-maze test, the angular error of 6-month-old PS19 mice was not different from that found in WT mice of 3-month-old PS19 mice (Fig. 4A). However, in the absence of the visual cue, path integration was significantly impaired (greater angular error) in 6-month-old, but not 3-month-old, PS19 mice; the deficit in the older PS19 mice was also greater than that observed in WT mice (Fig. 4B), indicating that high levels of phosphorylated tau and age contribute to navigational problems.

Importantly, the impaired capacity for path integration in 6-month-old PS19 was not due to accompanying deficits in hippocampus-dependent spatial learning and memory. This was shown by testing mice in the Barnes maze: although the PS19 mice were slower in learning the task (Fig. 4C), two-way ANOVA revealed that 6-month-old PS19 and WT mice travelled similar distances to reach the goal in the maze in the learning phase and did not differ in their error scores in the probe trial (Fig. 4D); PS19 and WT mice also did not differ in the time spent in the target quadrant (Fig. 4E). In summary, while 6-month-old PS19 mice do not display deficits in hippocampus-dependent spatial learning and memory, they exhibit significant impairments in EC-dependent path integration. Similar observations were made in older humans who show a decline in path integration despite the preservation of spatial memory.

#### Tau pathology in the EC of mutant mice and impaired path integration

**Figure 5:**
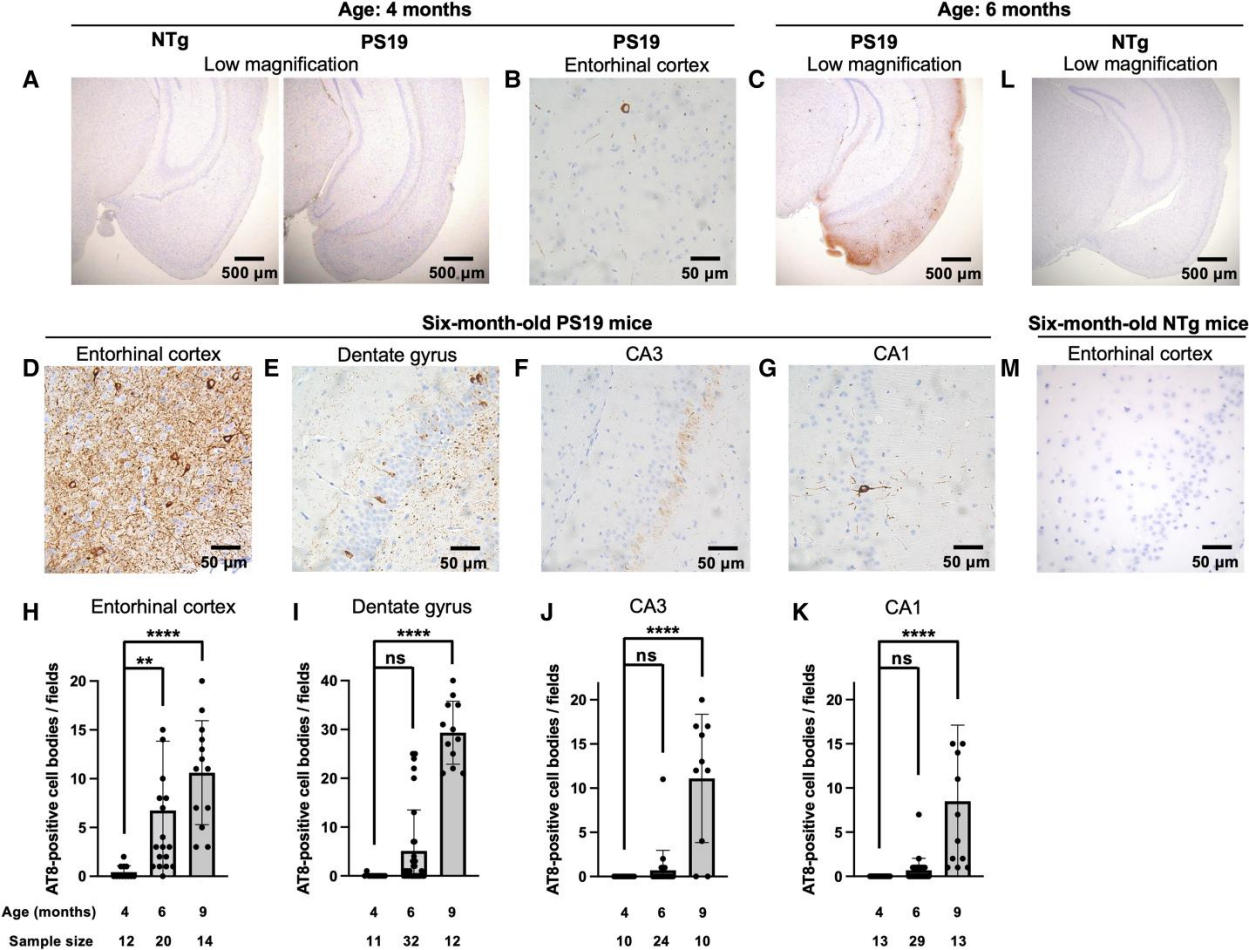
**Accumulation of phosphorylated tau in the EC of PS19 mice at 6 months of age.** (**A** and **C**) Paraffin sections from PS19 mice at 4, 6 and 9 months old and from their non-transgenic littermates (NTg) were immunostained with AT8 antibody, including the EC and hippocampus, and were observed under a low-magnification microscope in 4-month-old (**A**) and 6-month-old (**C** and **L**) NTg mice (left panel of **A** and **L**) and PS19 mice (right panel of **A** and **C**). (**B, D**–**G** and **M**) A higher magnification revealed AT8-positive cell bodies in the EC of PS19 (**B** and **D**) mice and NTg mice (**M**) at 6 months of age (**D** and **M**) and at 4 months of age (**B**), and in the dentate gyrus (**E**), CA3 (**F**) and CA1 (**G**) in PS19 mice at 6 months of age. (**H**–**K**) The AT8-positive cell bodies in the EC (**H**), dentate gyrus (**I**), CA3 (**J**) and CA1 (**K**) of PS19 mice were counted. The results are expressed as the mean ± SD; dots represent individual mean values. *P*-values were determined using one-way ANOVA and Dunnett's multiple comparisons test. *P < 0.05, **P < 0.01 and ***P < 0.001; ns, not significant. Detailed statistical analyses are provided in Supplemental Table 15.

To understand the relationship between deficits in path integration and pathological changes in PS19 mice, we investigated tau pathology using an AT8 phosphorylated tau antibody (Fig. 5). At 4 months of age, no difference in AT8 staining was observed between the WT and PS19 mice under low-magnification microscopy (Fig. 5A). At higher magnification, PS19 mice showed sparse positives in the neurites, but staining in the cell body was rarely observed in the EC (Fig. 5B). In mice at 6 months of age, we observed a relatively strong positive signal in the EC and a faint signal in the hippocampus (Fig. 5C). At higher magnification, some somatodendritic cells and most neurites in the EC were strongly stained with AT8 (Fig. 5D). In the hippocampus, very few somatodendritic cells and neurites showed positive staining (Fig. 5E–G). To compare age-related changes in the number of phosphorylated tau-positive cells, we next counted the AT8-positive neurons in the EC and hippocampus. The number of AT8-positive neurons in the EC was significantly increased at 6 and 9 months of age compared with the number in mice at 3 months of age (Fig. 5H). The number of AT8-positive neurons in the dentate gyrus and CA1 and CA3 of the hippocampus showed a similar trend. Six-month-old PS19 mice did not show a significant increase in the number of AT8-positive neurons compared with that in 4-month-old mice. However, at 9 months of age, the number of AT8-positive neurons was significantly increased (Fig. 5I-K). These results suggest that the accumulation of phosphorylated tau in the EC, but not the hippocampus, of PS19 mice at the age of 6 months caused path integration deficits without affecting hippocampal-dependent spatial cognition.

## Discussion

Early detection of the risk for developing Alzheimer’s disease is an important step in treating or preventing dementia, the most devastating symptom of Alzheimer’s disease. In the present set of experiments, we show that impaired path integration is an early behavioural sign of the accumulation of phosphorylated tau in the EC of both human and tauopathy mouse models.

Based on Braak and Braak’s^22,27^ studies on age-related development of NFTs and β-amyloid pathology, NFTs first appear in the EC and spread to the limbic system and the neocortex causing cognitive impairment over the next 30–40 years. Whereas β-amyloid accumulation occurs 10 years prior to the development of cognitive impairment,^28^ NFTs are formed by the replication of existing diffused NFT seeds after Braak Stage III,^29^ i.e. NFT seeds are already present in the neocortex during Braak Stages I and II. Indeed, we previously observed a significant increase in granular tau oligomers (NFT seeds) in the prefrontal cortex of individuals categorized as having Braak Stage I.^30^ The gradual accumulation of β-amyloid during Braak Stages I and II accelerates the replication of NFT seeds and promotes NFT formation beyond Braak Stage III. Clinical trials with therapies that target β-amyloid are currently being conducted in subjects with early Alzheimer’s disease or MCI ^31-33^ (albeit with limited success) when NFTs are likely to be already established in the limbic system and neocortex. Thus, the treatment or prevention of Alzheimer’s disease needs to start earlier—at Braak Stages I and II when NFTs are confined to the EC.

Our study focused on EC-dependent path integration since impairments in this function correlate strongly with a number of risk factors for Alzheimer’s disease such as the Cardiovascular Risk Factors, Aging and Dementia Study risk score,^21^ the apolipoprotein E ε4 allele^17,20^ and MCI in which reduced EC volumes are found.^16^

A key prediction of the present work is that individuals who exhibit path integration deficits above a certain threshold are expected to have NFTs in the EC (Supplemental Fig. 1). The expectation is based on previously published autopsy data that however comprised variable sample sizes and races, as well as individuals who experienced pathological events over varying durations. Although both deficits in path integration and NFT formation in EC are age-dependent events, we found a strong correlation between impaired path integration and NFT formation only when the behavioural deficit reached a certain threshold (>5 vm). Support for the notion that tau accumulation in the EC plays a causal role in disrupted grid cell activity comes from two different mouse lines. First, mice overexpressing aggregable human tau P301L exclusively in the EC exhibit NFT-like pathology and a loss of excitatory neurons in parallel with grid cell hypoactivity and reduced periodicity.^34^ Second, rTg4510 mice, a model expressing aggregation-prone tau, display decreased grid cell activity and loss of periodicity.^35^ Together, these observations suggest an inter-relationship between NFT in the EC and impaired path integration.

Our own experiments were conducted in PS19 mice, a widely used model of tauopathy that exhibits neuronal loss and NFT-like phosphorylated tau inclusions;^23^ the timing of the appearance of NFTs depends on the genetic background of the mouse and its rearing environment.^36^ Under our conditions, 6-month-old PS19 mice show impaired path integration, accompanied by abundant AT8-positive neurons in the EC. In fact, our results show that the angular error tends to increase with the number of AT8-positive cell bodies (Supplemental Fig. 4). Notably, AT8-positive neurons in the EC in 6-month-old PS19 mice did not react with the paired helical filament (PHF) tau conformation-specific tau antibody MC1 (Supplemental Fig. 5), indicating that grid cells inactivation may arise before PHF formation. It is worth noting here that the accumulation of phosphorylated tau is sufficient to trigger synaptic loss and decreased neural activity in the EC.^37^ In this context, we hypothesized that VR goggles can aid the early detection of individuals at risk for developing Alzheimer’s disease, opening a treatment window before tau aggregation.

Increased error distance (humans) and angular error (mice) reflect grid cell activity and serve as an index of impaired path integration ability (Supplemental Fig. 6). Here, we found that similar to aged humans, 6-month-old PS19 mice displayed increased angular error on the L-maze test in the absence of visual cues but had unimpaired spatial memory. While we still need to validate some of the parametric measurements and confirm the relationship between error distance and error angle thresholds and the extent of NFT, our data suggest that path integration is strongly indicative of EC dysfunction due to NFTs.

In conclusion, the present work shows that spatial learning and memory are maintained even when path integration is impaired. This suggests that path integration deficits due to the accumulation of phosphorylated tau in the EC are detectable before overt signs of impaired memory become manifest. We therefore posit that path integration is a more sensitive method to screen individuals who are likely to develop Alzheimer’s disease in the future. Since amyloid-β accumulation is a pathological feature of Alzheimer’s disease and an accelerating factor for spreading NFTs, screening for both, amyloid-β deposition and path integration—simpler and cheaper than screening for tau using PET—may be exploitable in the development of amyloid-β-targeted therapeutics.

Limitations of this work include the following: The results obtained in mice (association between the NFTs in EC and path integration) need to be further verified and validated in humans (e.g. association between individual path integration declines and Alzheimer’s disease biomarkers, tau PET, MRI). Longitudinal studies in larger-sized human cohorts are also required to track relationships between the course of Alzheimer’s disease and decline in path integration before the findings reported here can be applied as a diagnostic tool in humans.

## Supplementary Material

fcad359_Supplementary_Data

## Data Availability

The data that support the findings of this study are available from the corresponding author, upon reasonable request.
