# Supplementary material for: Path integration deficits are associated with phosphorylated tau accumulation in the entorhinal cortex
Source: Brain Commun. 2024 Feb 12;6(1):fcad359. doi: 10.1093/braincomms/fcad359 (PMC10859636; doi:10.1093/braincomms/fcad359)
Supplement: fcad359_Supplementary_Data [file fcad359_supplementary_data.zip › Supplemental video legends.pdf]

Supplemental video legends

The L-maze test was performed to investigate path integration in WT and PS19 mice. Mice were exposed to a conditioning phase in which the goal was set one hole to the left of the center. These representative videos show the behavior of the mice during the test phase. Six-month-old WT mice advanced toward the set goal (video 1), while Six-month-old PS19 mice moved in a different direction from the goal (video 2).
