# Supplementary material for: Path integration deficits are associated with phosphorylated tau accumulation in the entorhinal cortex
Source: Brain Commun. 2024 Feb 12;6(1):fcad359. doi: 10.1093/braincomms/fcad359 (PMC10859636; doi:10.1093/braincomms/fcad359)
Supplement: fcad359_Supplementary_Data [file fcad359_supplementary_data.zip › Supplementary_material.pdf]

Supplementary material

Path integration deficits are associated with phosphorylated tau accumulation in the entorhinal cortex

Riki Koike, Yoshiyuki Soeda, Atsushi Kasai, Yusuke Fujioka, Shinsuke Ishigaki, Akihiro Yamanaka, Yuta Takaichi, James K. Chambers, Kazuyuki Uchida, Hirohisa Watanabe, Akihiko Takashima

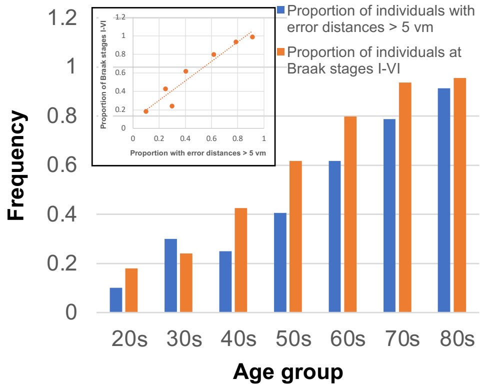

Supplemental Figure 1 The proportion of individuals exceeding 5 vm in the path integration VR-task correlated with the proportion of individuals with NFTs in the EC. This figure compares the proportion of individuals with error distances >5 vm in each age group (blue bars) to the proportion of NFTs (Braak stages I-VI) from brain autopsies at different ages, as reported by Braak and Braak [1] (orange bars). The correlation coefficient between the proportions across the age groups was 0.96 (*inset*).

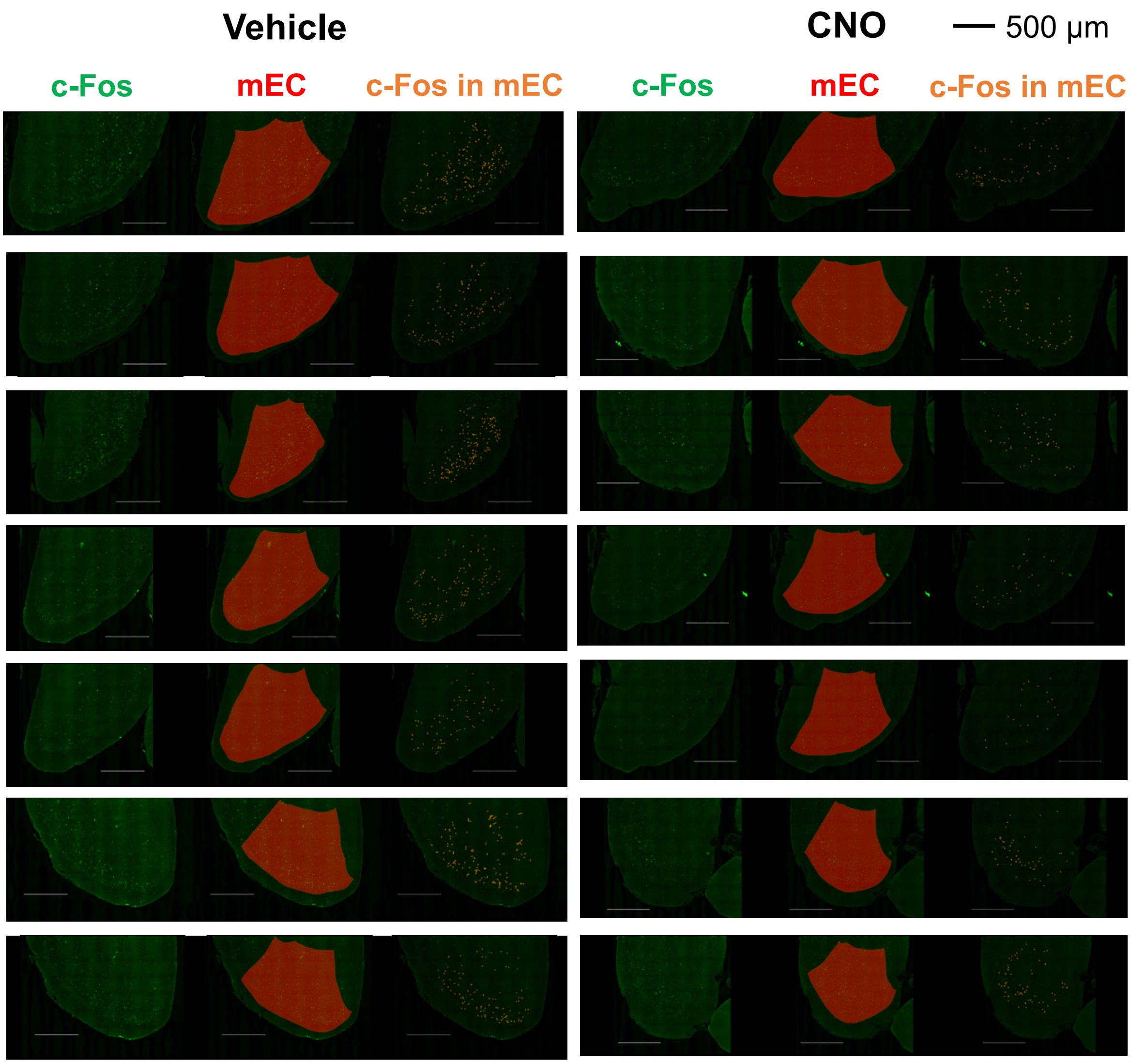

Supplemental Figure 2 Images of c-Fos staining including the entorhinal cortex 1 hr after the L-maze test in DREADD-expressing mice. One hour before the L-maze test, the mice were intraperitoneally injected with either CNO (1.0 µg/g of body weight) (CNO, n = 3) or an equivalent volume of 10% DMSO (10 µl/g of body weight) (vehicle, n = 3). The L-maze test was performed to examine the number of c-Fos positive neurons. Seven paraffin-embedded sections including the medial entorhinal cortex (mEC) obtained from each group were immuno-stained with c-Fos antibody as green fluorescence (left column). The region of mEC in the immuno-stained sections was positioned by a hand-drawn region-of-interest (red field in middle column). Merged images (orange) indicate c-Fos positive particles in the mEC (right column). Scale bar, 500 µm.

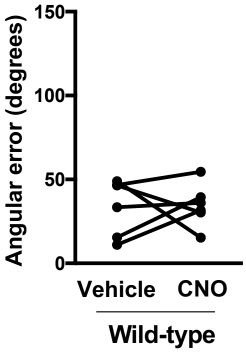

Supplemental Figure 3 An intraperitoneal injection of clozapine N-oxide (CNO) did not impair path integration in non-DREADD-expressing mice. Wild-type mice (n = 6) were intraperitoneally injected with either CNO (1.0 μg/g of body weight) or an equivalent volume of 10% DMSO (10 μl/g of body weight). One hour later, they were subjected to the L-maze test. A cross-over design was employed 1 day later to switch between the CNO- and vehicle-injected groups. There was no significant difference in the angular error ($p = 0.9254$). P value was determined using a paired t test.

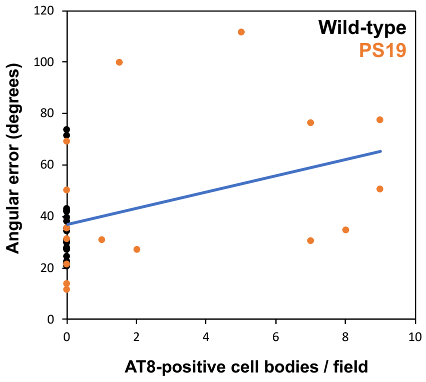

Supplemental Figure 4 Correlation of angular errors in the L-maze test with AT8-positive cell bodies in the entorhinal cortex. We examined the association between angular errors in the L-maze test without visual cues (Fig. 4B) and the number of AT8-positive neurons in the EC (3-month-old WT, n = 10; 3-month-old PS19, n = 8; 6-month-old WT, n = 9; 6-month-old PS19, n = 8). AT8-positive cell bodies in EC correlated positively with the angular error (Spearman's r = 0.3769; p = 0.0256). The brain samples were derived from the mice in Fig. 4B.

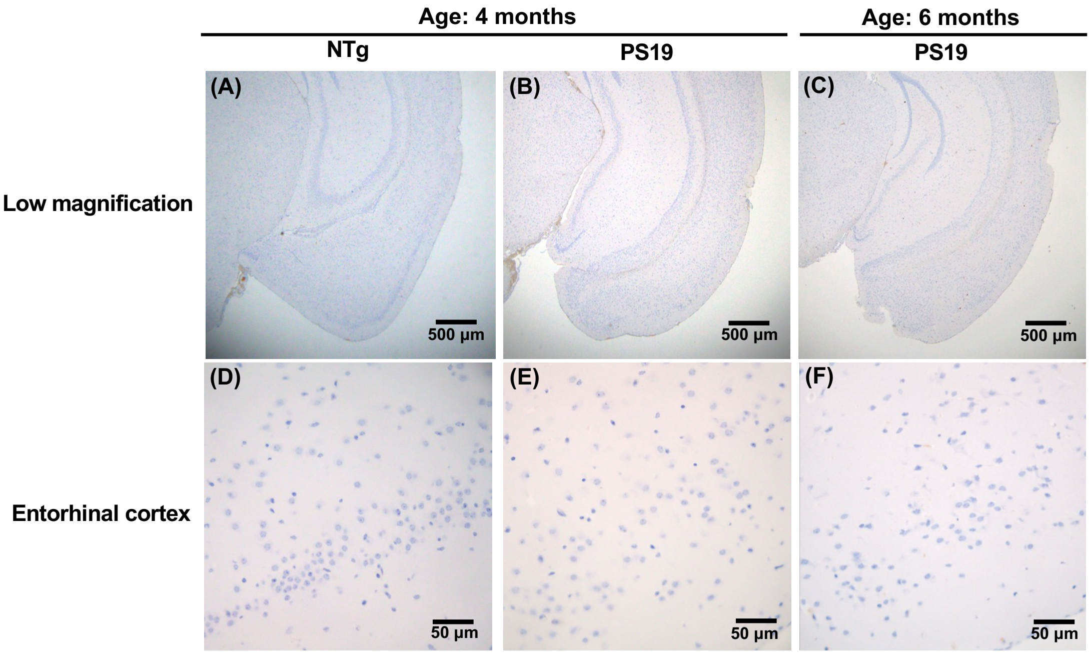

Supplemental Figure 5 Immunostaining of MC1-positive tau in PS19 mice overexpressing human P301S tau. Paraffin sections from PS19 mice and their nontransgenic littermates (NTg) were taken at 4 and 6 months old and immunostained with an MC1 antibody, which indicates a conformational change in tau [2]. AT8-immunostained sections were observed under both low (A, B, C) and high (D, E, F) magnification in NTg (A, D) and PS19 mice (B, C, E, F) at 4 (A, B, D, E) and 6 months of age (C, F).

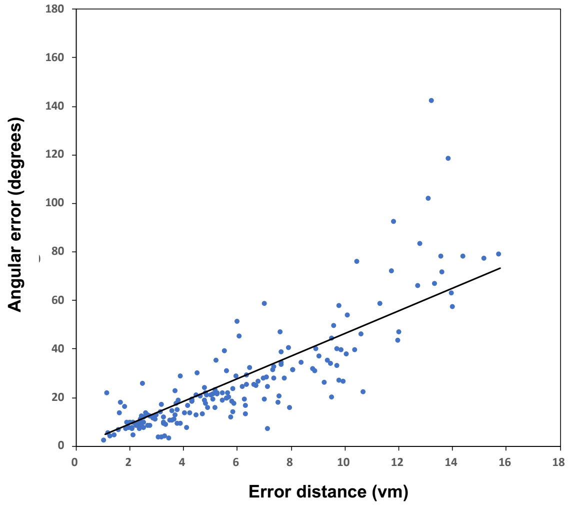

Supplemental Figure 6 Error distances correlated with angular errors in the path integration VR task. We investigated the relationship between two parameters (error distance and angular error), which were obtained from the human volunteers using the path integration test. Error distances well-correlated angular errors (r^2 = 0.8733). Previous reports have shown that these parameters in path integration can reflect grid cell activity [3,4].

Supplemental table 1: Information of statistical analysis in figure 1B.

Age	n	Statistical analysis		
20s	10	Repeated measures ANOVA	F (1.910, 17.19) = 3.546	P=0.0531
		Dunnett's multiple comparisons test	Trial 1 vs. trial 2	P=0.9991
			Trial 1 vs. trial 3	P=0.0829
			Trial 1 vs. trial 4	P=0.0222
			Trial 1 vs. trial 5	P=0.3294
30s	19	Repeated measures ANOVA	F (3.369, 60.64) = 6.256	P=0.0006
		Dunnett's multiple comparisons test	Trial 1 vs. trial 2	P=0.1097
			Trial 1 vs. trial 3	P=0.0205
			Trial 1 vs. trial 4	P=0.0019
			Trial 1 vs. trial 5	P=0.0006
40s	19	Repeated measures ANOVA	F (2.812, 50.62) = 5.582	P=0.0027
		Dunnett's multiple comparisons test	Trial 1 vs. trial 2	P=0.346
			Trial 1 vs. trial 3	P=0.0553
			Trial 1 vs. trial 4	P=0.0101
			Trial 1 vs. trial 5	P=0.0282
50s	36	Repeated measures ANOVA	F (2.810, 98.34) = 7.402	P=0.0002
		Dunnett's multiple comparisons test	Trial 1 vs. trial 2	P=0.0748
			Trial 1 vs. trial 3	P=0.1032

			Trial 1 vs. trial 4	P=0.0013
			Trial 1 vs. trial 5	P=0.0003
60s	34	Repeated measures ANOVA	F (2.824, 93.19) = 17.85	P<0.0001
		Dunnett's multiple comparisons test	Trial 1 vs. trial 2	P<0.0001
			Trial 1 vs. trial 3	P=0.0006
			Trial 1 vs. trial 4	P<0.0001
			Trial 1 vs. trial 5	P<0.0001
70s	31	Repeated measures ANOVA	F (2.804, 84.11) = 3.755	P=0.0158
		Dunnett's multiple comparisons test	Trial 1 vs. trial 2	P=0.059
			Trial 1 vs. trial 3	P=0.0304
			Trial 1 vs. trial 4	P=0.168
			Trial 1 vs. trial 5	P=0.0608
80s	22	Repeated measures ANOVA	F (3.089, 64.88) = 3.452	P=0.0205
		Dunnett's multiple comparisons test	Trial 1 vs. trial 2	P=0.4602
			Trial 1 vs. trial 3	P=0.0217
			Trial 1 vs. trial 4	P=0.0836
			Trial 1 vs. trial 5	P=0.44

Supplemental table 2: Information of statistical analysis in figure 1C.

Statistical analysis	Comparison		P value	Z value
Kruskal-Wallis test	20s-80s		0.0008	
Dunn's multiple comparisons test	20s (n=10)	30s (n=19)	>0.9999	0.3344
	20s (n=10)	40s (n=19)	>0.9999	0.6637
	20s (n=10)	50s (n=36)	>0.9999	0.4797
	20s (n=10)	60s (n=34)	>0.9999	0.8759
	20s (n=10)	70s (n=31)	0.0346	2.761
	20s (n=10)	80s (n=22)	0.0762	2.492

Supplemental table 3: Information of statistical analysis in figure 1D.

Statistical analysis	Comparison		P value	Z value
Kruskal-Wallis test	20s-80s		0.0048	
Dunn's multiple comparisons test	20s (n=10)	30s (n=19)	>0.9999	0.07817
	20s (n=10)	40s (n=19)	>0.9999	0.6393
	20s (n=10)	50s (n=36)	>0.9999	0.4655
	20s (n=10)	60s (n=34)	>0.9999	0.7078
	20s (n=10)	70s (n=31)	0.094	2.407
	20s (n=10)	80s (n=22)	0.194	2.132

Supplemental table 4: Information of statistical analysis in figure 2B.

Statistical analysis	Comparison		P value	Z value
Kruskal-Wallis test	20s-80s		<0.0001	
Dunn's multiple comparisons test	20s (n=10)	30s (n=19)	>0.9999	1.645
		40s (n=20)	>0.9999	0.8944
		50s (n=37)	0.6006	2.189
		60s (n=33)	0.3747	2.369
		70s (n=33)	0.0002	4.397
		80s (n=23)	0.0079	3.556
	30s (n=19)	40s (n=20)	>0.9999	0.9253
		50s (n=37)	>0.9999	0.4865
		60s (n=33)	>0.9999	0.7370
		70s (n=33)	0.0219	3.279
		80s (n=23)	0.4856	2.271
	40s (n=20)	50s (n=37)	>0.9999	1.563
		60s (n=33)	>0.9999	1.795
		70s (n=33)	0.0003	4.379
		80s (n=23)	0.0224	3.273
	50s (n=37)	60s (n=33)	>0.9999	0.3130
		70s (n=33)	0.0157	3.371
		80s (n=23)	0.6882	2.135
	60s (n=33)	70s (n=33)	0.0618	2.974
		80s (n=23)	>0.9999	1.811
	70s (n=33)	80s (n=23)	>0.9999	0.8843

Supplemental table 5: Information of statistical analysis in figure 2C.

P value by F-test	20s (n=10)	30s (n=19)	40s (n=20)	50s (n=37)	60s (n=33)	70s (n=33)	80s (n=23)
20s (n=10)							
30s (n=19)	0.8251						
40s (n=20)	0.3253	0.3228					
50s (n=37)	0.0039	0.0002	0.0034				
60s (n=33)	0.0127	0.0016	0.0226	0.3895			
70s (n=33)	0.0043	0.0002	0.0041	0.9679	0.4241		
80s (n=23)	0.0128	0.0019	0.0247	0.5067	0.9202	0.5381	

Supplemental table 6: Age and number of subjects in PS19 mouse's experiments.

	L-maze test in Fig. 4A			L-maze test in Fig. 4B			Barnes maze			Immunohistochemistry		
	Mean age (month)	Female no. (%)	Male no. (%)	Mean age (month)	Female no. (%)	Male no. (%)	Mean age (month)	Female no. (%)	Male no. (%)	Mean age (month)	Female no. (%)	Male no. (%)
**3-4 months**	4.1±0.7	11(61.1)	7(38.9)	4.0±0.4	12(66.7)	6(33.3)	NA	NA	NA	4.0±0.0	1 (50.0)	1 (50.0)
**6 months**	7.0±0.4	7(38.9)	11(61.1)	7.0±0.4	7(38.9)	11(61.1)	6.6±0.6	11(61.1)	7(38.9)	6.5±0.5	3 (75.0)	1 (25.0)
**9 months**	NA	NA	NA	NA	NA	NA	NA	NA	NA	9.0±0.0	0 (0.0)	4 (100.0)

Age expressed as mean ± SD.

Supplemental table 7: Age and number of subjects in DREADD experiments.

	Mean age (month)	Female<br>no. (%)	Male<br>no. (%)
**DREADD**	4.1±0.1	0(0)	6(100)

Age expressed as mean ± SD.

Supplemental table 8: Information of statistical analysis in figure 3D left panel.

Statistical analysis	Comparison		P value	t value	degrees of freedom
T-test	Vehicle (n=7)	CNO (n=7)	0.0016	4.040	12

Supplemental table 9: Information of statistical analysis in figure 3D right panel.

Statistical analysis	Comparison		P value	t value	degrees of freedom
Paired T-test	Vehicle (n=6)	CNO (n=6)	0.0034	5.213	5

Supplemental table 10: Information of statistical analysis in figure 4A.

Statistical analysis	Comparison		P value	F value	
Two-way ANOVA	interaction		0.5409	F (1, 32) = 0.3821	
	genotype		0.3441	F (1, 32) = 0.9224	
	age		0.2413	F (1, 32) = 1.425	
Tukey's multiple comparisons test	**Comparison**		**P value**	**q value**	**degrees of freedom**
	WT 3mo	WT 6mo	0.9726	0.6106	32
	WT 3mo	PS19 3mo	0.9949	0.3423	32
	WT 3mo	PS19 6mo	0.4359	2.154	32
	WT 6mo	PS19 3mo	0.9984	0.2334	32
	WT 6mo	PS19 6mo	0.6824	1.579	32
	PS19 3mo	PS19 6mo	0.6218	1.719	32

Supplemental table 11: Information of statistical analysis in figure 4B.

Statistical analysis	Comparison		P value	F value	
Two-way ANOVA	interaction		0.0373	F (1, 32) = 4.722	
	genotype		0.068	F (1, 32) = 3.569	
	age		0.0267	F (1, 32) = 5.393	
Tukey's multiple comparisons test	**Comparison**		**P value**	**q value**	**degrees of freedom**
	WT 3mo	WT 6mo	0.9995	0.1582	32
	WT 3mo	PS19 3mo	0.9971	0.2839	32
	WT 3mo	PS19 6mo	0.0268	4.211	32
	WT 6mo	PS19 3mo	0.9898	0.4331	32
	WT 6mo	PS19 6mo	0.0344	4.062	32
	PS19 3mo	PS19 6mo	0.0245	4.265	32

Supplemental table 12: Information of statistical analysis in figure 4C.

Statistical analysis	Comparison		P value	F value	
Repeated measures two-way ANOVA	interaction		0.3436	F (2, 32) = 1.105	
	genotype		0.3369	F (1, 16) = 0.9802	
	day		0.0158	F (2, 32) = 4.735	
Bonferroni's multiple comparisons test	**Comparison**		**P value**	**t value**	**degrees of freedom**
	WT day1	PS19 day1	> 0.9999	0.2832	48
	WT day2	PS19 day2	> 0.9999	0.1944	48
	WT day3	PS19 day3	0.2609	1.747	48

Supplemental table 13: Information of statistical analysis in figure 4D.

Statistical analysis	Comparison		P value	t value	degrees of freedom
T-test	WT (n=8)	PS19 (n=10)	0.3265	1.012	16

Supplemental table 14: Information of statistical analysis in figure 4E.

Statistical analysis	Comparison		P value	t value	degrees of freedom
T-test	WT (n=8)	PS19 (n=10)	0.4772	0.7279	16

Supplemental table 15: Information of statistical analysis in figure 5H-5K.

Region	Statistical analysis	Comparison		P value
Entorhinal cortex	One way ANOVA	F (2, 43) = 12.20		P<0.0001
	Dunnett's multiple comparisons test	4 months (n=12)	6 months (n=20)	0.0053
		4 months (n=12)	9 months (n=14)	<0.0001
Dentate gyrus	One way ANOVA	F (2, 52) = 61.85		P<0.0001
	Dunnett's multiple comparisons test	4 months (n=11)	6 months (n=32)	0.0843
		4 months (n=11)	9 months (n=12)	<0.0001
CA3	One way ANOVA	F (2, 41) = 30.20		P<0.0001
	Dunnett's multiple comparisons test	4 months (n=10)	6 months (n=24)	0.8288
		4 months (n=10)	9 months (n=10)	<0.0001
CA1	One way ANOVA	F (2, 52) = 20.99		P<0.0001
	Dunnett's multiple comparisons test	4 months (n=13)	6 months (n=29)	0.8464
		4 months (n=13)	9 months (n=13)	<0.0001

List of raw data in figures 1B-1D, 2B, 2C + Supplemental figure 6.

Age group	Test name	Subject No.	Age (years)	Sex	Figure 1B					Figure 1C	Figure 1D	Figure 2B and C		Supplemetal figure 6	
					Distance traveled (vm)					Error score (vm)	Time in target quadrant (s)	Error distance (vm) (trial 2-3)	Probability	Error distance (vm) (all)	angular error (degrees) (all)
					Trial 1	Trial 2	Trial 3	Trial 4	Trial 5						
20s	POC3	075	20	female	27.989008	11.904429	13.203556	12.338911	12.463487	421.16424	47.5	2.814454824	0.2715963	2.697660494	8.253982172
	POC3	072	21	female	45.305284	51.766081	50.406189	52.639815	43.27012	375.36925	47	1.729931078	0.2217705	3.483397945	3.098771534
	POC3	077	21	female	29.98329	39.439128	16.650362	12.864523	18.867154	627.27661	41.5	2.460976997	0.2700798	2.552998548	9.289384096
	POC3	051	24	female	35.536146	17.29777	11.943753	21.902456	11.931081	733.27671	39	0.39124342	0.0808434	1.457111522	4.343121823
	POC3	079	28	female	35.351591	16.455815	21.805643	15.36997	12.667114	649.94893	41	5.082173743	0.0700727	4.346722224	18.93558862
	POC3	054	20	male	46.760251	12.867909	15.981392	16.57231	14.508638	773.31428	41	1.945390889	0.2412218	1.707104728	17.51817264
	POC3	073	20	male	35.211418	35.936899	21.453774	13.70656	13.537422	603.11712	39	1.8031457	0.2287543	2.399955268	6.801500346
	POC3	070	21	male	12.172003	24.965209	11.665802	11.424499	12.913719	454.50298	46	4.957256475	0.0803817	5.475982995	21.40959541
	POC3	041	28	male	16.53296	34.497244	22.346032	12.424927	16.039963	364.9818	50	2.154023321	0.2563198	3.853352282	18.43214615
	POC3	074	29	male	24.444733	53.423454	23.283036	17.6274	55.874164	443.86013	43.5	3.377098389	0.2428961	3.60671656	10.13507803
30s	P0C3	032	30	female	39.783096	20.766931	13.882162	13.354245	21.35677	951.18718	0	2.629761422	0.1519147	3.324308408	3.941060219
	P0C3	005	31	female	17.867817	23.807614	15.48255	17.032514	12.550852	513.52206	50	5.176539739	0.2094497	4.071623786	13.38657899
	P0C3	010	32	female	37.963524	21.909975	20.80164	23.351469	27.993898	669.59257	38.5	6.670707175	0.0767865	9.540949012	19.78632585
	P0C3	003	35	female											
	P0C3	013	36	female	25.633375	13.146774	31.411784	12.545409	17.467763	530.88076	45	2.465066029	0.1362372	2.144774622	4.3193292
	P0C3	020	36	female	19.280996	12.159784	12.063912	23.733875	30.895347	1272.5285	0	4.241510535	0.2506508	4.74936811	12.80211502
	P0C3	030	36	female	37.778592	24.802453	30.001383	24.21676	16.570245	647.90445	43	5.379306468	0.1924814	5.796286387	11.5851967
	P0C3	011	38	female	12.798101	17.951365	23.543793	13.290372	19.491502	362.57382	50	2.532514601	0.1426365	3.016689449	12.56544039
	P0C3	016	39	female	25.654419	42.578914	15.041872	18.421156	20.329513	743.04738	47	6.248312317	0.1115048	8.844641613	31.41046402
	POC3	052	33	female	36.103057	12.260111	21.421692	18.661207	15.131435	499.59768	43	3.899005449	0.2455413	3.507345777	10.19995978
	POC3	053	39	female	39.46757	24.703069	13.118644	34.658433	17.078758	653.97516	39.5	6.245248553	0.1117781	6.639798111	25.05604455
	P0C3	027	30	male	18.232197	12.992444	16.303125	13.247273	12.258728	521.79284	43	2.444722165	0.1343166	1.987679587	7.226054357
	P0C3	009	32	male	39.875729	31.400038	18.615713	11.812429	21.36885	374.98556	44	6.123465781	0.1228346	6.482706159	31.97565251
	P0C3	017	32	male	26.399945	42.00844	42.488061	13.152218	14.523253	851.65293	33.5	3.238992403	0.2070918	2.39607098	8.129743948

	P0C3	002	33	male	52.125506	15.790555	23.873309	11.259925	28.845085	369.81318	51	5.8006687	0.1533193	4.870835314	17.3134341
	P0C3	001	34	male	33.497568	27.415338	30.021027	23.710262	23.47146	570.60524	48.5	4.181731428	0.250586	3.815443451	8.925139177
	P0C3	023	35	male	45.730886	10.897012	19.213052	28.987084	13.603959	514.00316	41.5	3.38868493	0.2185177	2.718123286	12.3559717
	P0C3	008	38	male	12.630518	13.115407	17.278234	26.270621	13.356775	553.9814	39.5	2.602009587	0.1492639	2.548686435	7.07564451
	POC3	057	37	male	25.657161	19.88909	21.475658	11.933903	12.668614	261.16767	50	1.761006382	0.0757943	1.282935426	3.980624237
	POC3	050	39	male	37.43814	48.104025	16.40626	12.091367	14.580411	383.55457	48.5	5.199510252	0.2076239	5.616277247	19.39847158
40s	POC3	035	42	female	12.098262	23.15064	33.357204	13.238273	28.403254	1355.9352	0	0.532831427	0.0678962	1.064902962	1.88106128
	POC3	039	42	female	29.598114	17.873936	19.006727	13.271933	13.655112	443.4758	46.5	3.261820848	0.196888	3.305442895	8.824267134
	POC3	018	44	female	38.534034	27.49439	17.431553	11.404269	11.379062	349.43356	46.5	2.759511173	0.1857672	4.335129763	17.89111933
	POC3	014	45	female								2.387658931	0.1709547	2.132279585	7.081727335
	POC3	019	45	female	19.885818	27.863254	13.132382	18.450882	27.417675	992.82051	0	5.345472776	0.1290041	5.186513269	23.06998765
	POC3	006	47	female	37.51127	22.532717	12.479482	12.500096	19.277421	448.15507	46.5	6.630448562	0.0583069	5.721187627	19.68846952
	POC3	024	48	female	37.621552	30.273234	19.969367	12.171252	23.815531	597.42937	39.5	2.914917208	0.1903996	3.910487809	8.992421981
	POC3	026	48	female	25.809168	34.681726	16.427845	12.257612	11.80512	374.5	48.5	2.714471296	0.1842408	2.792844245	8.09601069
	POC3	028	49	female	42.579398	12.585691	26.450507	12.936588	21.380101	935.61964	31.5	3.788024079	0.1957524	3.296154233	9.288058416
	POC3	087	40	female	25.254558	13.526476	19.262888	25.53495	20.230617	659.22171	44.5	3.242799273	0.1966776	3.271316496	11.40474897
	POC3	007	40	male	34.271979	23.939864	35.731803	20.134017	22.106151	1121.7351	19.5	6.230389599	0.0779902	5.883886361	23.51174614
	POC3	015	41	male	27.678146	13.072385	17.596632	13.811449	24.195055	535.59433	43	0.847002157	0.0842706	2.123490345	6.910500103
	POC3	036	42	male	35.265106	28.514224	29.171721	28.051119	20.098976	855.19985	24.5	8.043182971	0.0152261	5.83427038	18.28059766
	POC3	012	44	male	38.111565	12.882911	29.073114	13.124922	14.295919	442.18791	48	4.341193183	0.1807646	4.847433481	21.71968754
	POC3	021	44	male	44.352784	18.584897	20.620373	16.447017	12.913905	493.85169	47.5	1.673784977	0.1324712	1.861688104	16.1437909
	POC3	029	44	male	19.056396	33.494792	15.812157	13.748991	11.772012	357.68002	48	1.158554913	0.1019276	3.203334314	3.505173382
	POC3	004	46	male	31.795217	34.615823	25.770927	26.094153	30.734591	582.57392	41	1.880283733	0.1444666	1.664570067	13.32887464
	POC3	022	46	male	23.830949	36.796766	12.464541	22.912338	20.928707	501.55972	45	3.487568948	0.1980484	2.630542647	13.3779721
	POC3	037	47	male	16.314356	15.133738	12.380313	12.314833	20.386476	637.93948	44.5	2.782172635	0.1865048	2.383062511	9.273133313
	POC3	025	49	male	12.325014	26.795757	33.939954	40.143054	25.933398	582.73067	39.5	5.584307392	0.1147816	5.203253565	15.62526254
50s	POC3	033	52	female	26.711061	37.213952	20.533911	16.71812	25.793789	720.19742	43	7.133937238	0.0921137	7.174763488	24.07692915
	POC3	034	52	female								3.961689777	0.0962887	3.811388835	14.52321106
	POC3	038	57	female	47.788597	25.535187	25.060142	27.067899	16.524139	523.18778	44.5	1.289798376	0.0597095	1.59959196	6.572054169
	POC3	042	52	female	28.254139	14.438283	18.257026	20.175924	13.055378	585.52345	41.5	3.554743025	0.0922941	3.695231641	10.93574631

	POC3	044	56	female	34.845086	32.30347	10.834351	26.125399	37.88589	607.4666	37	3.547860422	0.0922193	4.251720661	13.41166879
	POC2	36	50	female	33.520487	13.093036	13.1037	17.382946	16.227396	403.81224	50.5	1.747723967	0.0670099	4.661565842	20.12696834
	POC2	4	51	female	35.133305	40.195827	42.572636	28.84369	29.346943	352.51986	53.5	6.272868929	0.0995556	5.252629773	34.94958041
	POC2	63	51	female	20.768382	16.480115	6.1963298	10.016168	5.8982508	1330.9189	0	4.746501261	0.101312	7.005722884	27.54035809
	POC2	85	51	female	47.431889	23.804647	23.991325	18.134001	15.083738	847.39305	29.5	6.128052419	0.1003814	6.358945588	28.96144381
	POC2	84	52	female	57.839772	12.238004	30.883868	22.368942	13.685722	565.83766	45.5	9.392994748	0.0595392	8.948917185	39.62306729
	POC2	37	53	female	13.199713	27.932981	13.148892	12.532861	23.969466	742.90614	32	4.886472762	0.1017993	5.13838161	19.14749593
	POC2	40	53	female	36.086172	12.47349	23.984705	19.742709	22.407827	400.47052	49.5	2.907184893	0.0843545	2.464958889	11.83342914
	POC2	64	53	female	38.9091	42.351624	25.975682	32.566234	12.137712	269.25737	51.5	1.065436328	0.0561443	2.938541944	11.78736505
	POC2	55	54	female	42.870816	30.358728	26.111686	34.051749	20.499816	473.25764	48.5	5.94418377	0.1012376	4.878888459	20.69600143
	POC2	3	55	female	19.733291	25.375111	34.162285	24.522232	33.348729	831.22829	40.5	14.06565031	0.0082925	13.24776954	142.1384815
	POC2	54	55	female	38.274446	39.494927	37.027963	51.944598	37.593134	1246.3067	16	3.718128575	0.0940009	6.377119805	24.94656002
	POC2	71	55	female	24.1471	17.821632	19.963131	14.637207	19.638228	577.75194	37.5	4.911445592	0.1018727	4.333822794	17.97008187
	POC2	38	56	female	55.83453	12.548612	11.947438	13.19137	23.369231	438.35602	51.5	10.91014095	0.0367648	7.359130278	30.9710724
	POC2	70	56	female	19.947084	24.172132	29.953514	27.473367	24.260488	1209.432	2	14.33794176	0.0070714	10.63616464	45.76391021
	POC2	39	57	female	37.809871	13.026651	12.065587	12.388197	19.004788	821.79647	35	15.46385392	0.0034744	15.76487854	78.96654991
	POC2	81	58	female	14.903377	31.239655	24.080365	14.133039	21.660356	334.98868	7.5	2.160106039	0.0734699	3.605780481	14.30504613
	POC3	040	54	male	27.293467	33.663687	44.886129	29.320216	28.226654	366.40669	50.5	3.47713311	0.0914378	2.557151958	11.66258943
	POC3	068	51	male	30.04662	11.928433	12.178866	11.795354	17.45965	541.64328	48.5	8.63087989	0.0716287	7.150608471	6.756912042
	POC3	045	54	male	27.293467	33.663687	44.886129	29.320216	28.226654	366.40669	50.5	3.47713311	0.0914378	2.557151958	11.66258943
	POC3	061	56	male	24.882121	11.956061	22.679734	21.589761	12.98276	414.33683	45	0.985093416	0.0548757	2.894246939	10.9531447
	POC2	2	51	male	40.488954	23.830188	12.863525	20.932163	10.725134	341.58935	50	5.244902174	0.1024524	3.733990254	22.27333089
	POC2	72	53	male	40.477147	35.079684	24.701474	17.584582	24.289275	496.97521	42.5	1.309650487	0.0600261	1.191552135	21.72259789
	POC2	83	53	male	12.273182	24.7861	11.893972	16.431049	12.911361	429.25685	46	2.128781156	0.0729868	2.040833461	9.608875898
	POC2	82	56	male	13.086063	16.177978	21.701511	21.286707	12.883994	604.28256	40.5	4.384200875	0.0994623	4.817227527	18.58261509
	POC2	86	56	male	15.930908	39.677389	35.024793	16.321467	26.152877	774.24701	26	2.326162819	0.0760025	2.289588751	8.055624701
	POC2	68	57	male	11.555559	24.456313	15.045593	22.197247	21.38339	826.1018	36.5	1.659013419	0.0656	1.929771115	9.539682932
	POC2	79	57	male	39.363241	55.578426	52.385837	24.024305	14.929198	784.85308	36	1.831618953	0.0683385	4.937783114	15.53082902
	POC2	80	57	male	50.95608	18.451292	18.98136	16.798345	24.480652	649.55461	31.5	5.305772994	0.1024774	5.113663063	20.9737938
	POC2	35	58	male	29.138524	14.71139	23.174975	13.354328	11.76274	592.30505	43	12.29108621	0.0207736	13.62704355	71.32852815

	POC2	1	59	male	31.04981	13.534523	43.418256	33.300769	13.311496	973.0517	7.5	7.508004889	0.0877093	8.394009178	34.07903127
	POC2	61	59	male	12.588788	11.392864	38.710626	27.006158	12.072984	343.94867	48	5.791619745	0.1017811	4.547233931	29.99539701
	POC3	089	50	male	35.846657	11.055255	15.865307	11.778194	22.865903	460.43563	46.5	2.936268161	0.0847463	3.708954941	12.34791461
60s	POC3	031	60	female	43.898958	23.013208	44.8618	43.153461	41.123347	705.10024	45.5	3.096751211	0.0949773	5.467434966	18.45034473
	POC3	062	60	female	24.20059	11.615765	12.686204	19.621743	12.284351	544.54474	40	4.803820819	0.1175762	4.133869451	7.426097521
	POC3	043	61	female	38.717151	24.959599	16.905532	16.40424	16.62454	718.83557	38	3.061019601	0.0942916	2.487277988	7.961720202
	POC3	047	61	female	45.066589	20.602286	22.029497	18.183746	18.293215	809.08627	38	12.90232197	0.0093679	9.987096931	26.44199353
	POC3	065	67	female	28.916439	14.233701	16.967014	19.726775	11.629308	992.80659	27.5	8.227728187	0.0823941	6.230103279	24.0311312
	POC2	13	61	female	31.86925	54.35715	54.096092	44.455384	27.454489	1205.1306	0				
	POC2	7	62	female	31.142202	15.550207	30.319728	19.46449	18.009499	592.55324	44	1.990165666	0.0719795	3.915574021	28.51230451
	POC2	8	62	female	38.957763	48.785769	25.959393	40.452439	12.184808	256.10463	52	6.160167056	0.1157379	6.73278449	24.70592794
	POC2	9	62	female	31.21238	21.82035	25.787307	17.629337	17.22353	349.11563	48	2.172835361	0.0759198	5.859019028	13.60820947
	POC2	14	62	female	50.780698	18.961198	16.800649	13.92758	14.762997	453.44749	46	3.815706395	0.1072567	5.987320312	28.41867945
	POC2	19	62	female	56.906684	38.330689	12.069857	18.221252	12.602798	360.5363	49	1.49590823	0.06139	2.527096907	25.6571769
	POC2	57	64	female	25.600798	19.952947	22.360595	20.586862	27.249039	432.62715	46.5	2.348296898	0.079684	2.161970514	9.586171413
	POC2	10	65	female	32.330567	32.432457	25.988751	33.1945	12.811865	382.5688	49.5	2.910849663	0.0913497	4.18584972	16.45031774
	POC2	11	66	female	23.038813	25.775788	18.538603	21.320113	19.402672	311.15494	50.5	6.796066976	0.1085734	6.346184197	16.54044007
	POC2	74	66	female	33.667978	12.378343	28.612889	21.951484	19.417304	295.32169	51	2.922402685	0.0915793	2.434185345	10.81612619
	POC2	6	67	female	38.371966	18.125619	25.392307	25.307289	12.90637	540.92471	46.5	0.974700193	0.050695	3.77062404	17.17125919
	POC2	12	68	female	32.23636	19.504976	34.221158	38.851166	34.692435	838.54727	32	8.515462515	0.0762481	7.666970543	38.25716414
	POC2	73	69	female	21.025188	28.588449	30.945084	21.363831	17.189067	447.13785	45.5	6.543592553	0.1118452	9.539426725	43.97478579
	POC2	76	69	female	29.584462	30.25061	33.758928	28.712474	24.852907	1391.7518	0	5.397049197	0.1191507	4.812851715	23.64456869
	POC3	067	61	male	24.171615	13.960925	27.453762	13.052567	15.246589	1025.7027	0	3.811636222	0.1071968	4.49927461	12.62131872
	POC3	066	64	male	39.97309	22.691755	29.356532	20.199001	12.018882	1443.9039	0	5.174371335	0.1189944	5.640245731	19.32118583
	POC3	046	68	male	23.780977	34.269599	23.128355	26.917314	23.403585	437.80465	46.5	6.506975275	0.1122748	7.581248555	20.39484398
	POC3	049	68	male	35.397023	12.991268	16.518	21.63041	20.207213	425.64961	47.5	6.969593512	0.1060301	5.66417785	21.55279918
	POC2	69	60	male	34.79103	37.123451	25.832237	17.68055	19.223626	505.12594	45	2.575100521	0.0844829	1.866476052	6.732788427
	POC2	15	61	male	34.548911	29.267916	23.168884	22.287392	28.869921	1013.5126	0	7.845814489	0.0902877	10.11238975	53.69317494
	POC2	20	61	male	22.787098	12.121481	17.794618	13.666412	19.80053	522.59428	44.5	2.235972121	0.0772778	6.008186841	51.14925097
	POC2	16	62	male	39.515663	26.265896	12.5529	18.010747	12.702644	820.88796	37	11.55094808	0.0214595	9.896576444	39.28894842

	POC2	21	62	male	25.729845	17.629487	17.744141	19.365761	17.341058	647.2097	37.5	2.563084418	0.0842312	5.266755001	21.40227411
	POC2	62	64	male	43.127846	16.903106	18.029547	16.643171	12.717242	498.29436	48	8.803289672	0.0700392	8.10153991	31.2577292
	POC2	18	65	male	57.77673	36.090955	15.019264	15.381343	14.29812	917.27205	41.5	4.822869155	0.1176838	6.793348642	26.31733557
	POC2	59	66	male	22.044698	17.665964	15.278262	18.795434	15.015055	453.57091	42.5	4.801177903	0.1175609	5.052143719	20.77325342
	POC2	5	67	male	43.073869	11.828544	22.681433	31.649209	29.36811	1167.3812	0	13.53245171	0.00602	13.38384117	66.77118835
	POC2	17	69	male	50.925992	43.723158	22.048525	11.595309	18.533554	647.84872	40.5	9.771032228	0.0498627	9.496118063	33.66988608
	POC3	080	64	male	37.73099	11.918249	46.086257	16.750794	18.510395	627.59345	41.5	1.509346423	0.061674	1.237721289	5.251427967
70s	POC3	055	70	female	38.567901	18.120128	12.102514	36.245956	19.383535	1393.1357	10.5	7.670587024	0.1018456	9.395371264	34.91276245
	POC3	076	71	female								1.667995579	0.0233761	3.100250613	3.393571129
	POC3	048	72	female	39.229653	24.741675	21.695873	19.495189	16.284286	537.43978	40.5	9.555765165	0.0982001	9.258456112	25.99137912
	POC3	069	75	female	39.104828	21.30351	31.8361	13.856182	43.970843	827.98182	30.5	3.355586649	0.0451365	3.377058984	8.503928009
	POC3	063	76	female								5.045344871	0.0720332	4.351841267	18.6688675
	POC2	30	70	female	2.5060252	18.473911	16.644276	19.309883	4.9299668	1301.1569	0	13.94264965	0.0358542	13.12953909	101.4972644
	POC2	23	72	female	22.939443	23.881366	48.052909	52.352983	50.971496	889.43612	29	11.53384498	0.0731481	11.75520197	71.716601
	POC2	41	72	female	21.089729	23.930336	26.266515	29.035999	16.543638	695.25844	37	11.03308629	0.0807901	12.82393721	83.25115658
	POC2	43	76	female	36.484478	45.695072	29.423356	27.473932	19.629705	1156.3863	15.5	2.541275066	0.0336511	2.25235201	9.133174999
	POC2	25	77	female	22.074663	17.838856	25.762656	28.827812	39.746775	1236.3082	0	8.868155663	0.1023008	9.824547797	57.65244953
	POC2	26	78	female	32.792488	20.85159	43.021409	23.666481	23.93863	1070.6397	10	8.63722842	0.1029809	9.60859123	49.38760475
	POC2	75	78	female	51.425499	33.458665	41.476413	49.702546	34.146125	1042.9271	17.5	13.68239132	0.0394586	14.04227721	57.14937248
	POC3	083	77	female	32.988825	22.695111	13.909234	17.546492	12.892535	495.92573	48.5	9.432767396	0.099152	7.372410937	27.75697627
	POC3	056	74	male	33.469188	16.877113	36.084706	21.106824	44.491887	1026.0773	19.5	5.498356139	0.0790349	6.28843336	18.75642975
	POC3	059	74	male	22.141521	34.186308	38.176109	42.143565	40.286516	1630.5854	0	11.7164222	0.0702509	10.40639243	39.33090663
	POC3	060	76	male	10.991582	45.386862	19.743681	27.696363	17.315021	590.98187	43	4.055766151	0.0560709	3.160406132	13.77877312
	POC3	064	77	male	53.696114	11.937425	12.542597	36.600341	19.736252	1349.7799	0	7.89209256	0.1026714	9.748326384	39.63645533
	POC2	28	70	male	56.826465	17.668314	14.607533	16.937992	16.207419	436.54959	47	8.677522256	0.1028884	6.326016583	12.73789785
	POC2	34	70	male	23.503776	17.642583	25.479548	30.408784	35.283768	946.37801	28.5	6.037863728	0.086696	7.648452189	34.23162682
	POC2	46	70	male	8.9541536	24.392804	23.558942	29.954592	17.920474	379.34827	46.5	6.993177769	0.0973528	5.292732059	21.03244657
	POC2	47	70	male	48.602082	47.618729	29.541291	55.534929	51.920915	846.1382	32	9.536726725	0.0983533	11.83565512	92.33939526
	POC2	24	71	male	56.885978	40.180793	26.080942	33.896029	20.439185	894.37284	26	15.01289296	0.0230529	13.87689198	118.2227237
	POC2	31	71	male	54.933475	25.737709	12.556947	13.862456	24.845894	434.18641	47	2.656053083	0.0351677	2.798414186	11.99384538

	POC2	32	71	male	11.169028	36.382737	17.207611	15.95634	31.114974	1121.9295	23.5	14.84382311	0.0248454	11.98186838	43.40464852
	POC2	77	71	male	42.816279	31.199735	32.207821	21.860311	20.245582	507.28688	48	10.35951254	0.0899261	11.31994443	58.191018
	POC2	33	72	male	2.9661916	3.0359345	14.307004	30.341028	20.354302	1213.2768	8	4.969605367	0.0708294	7.021185339	58.17654508
	POC2	45	72	male	51.079389	21.997433	33.789339	24.812473	35.320776	701.24048	42.5	9.516644843	0.0985127	7.923691678	40.29932522
	POC2	22	73	male	51.492768	34.113752	11.946431	34.614736	12.502021	538.7953	43.5	9.138529673	0.1010499	8.092023783	31.08145665
	POC2	44	73	male	47.176116	31.52077	33.103225	29.197318	30.40713	699.66446	40.5	7.114483909	0.0983648	9.054674648	36.75539412
	POC2	78	75	male	43.022329	39.737618	30.457786	17.760198	26.364986	766.75788	30	10.76563616	0.0846086	10.08096185	37.58629785
	POC2	42	77	male	27.922242	26.615561	10.753581	22.995981	14.115454	1812.1315	0	15.2436353	0.0207491	15.19998718	77.1819818
	POC2	29	79	male	49.33551	34.389259	30.603416	19.481059	19.535073	769.66744	37	5.274292679	0.0756206	7.657356967	33.12091504
	POC3	081	75	male	35.771859	19.408261	19.122955	15.575747	21.673619	600.89998	43	2.451646452	0.0324925	2.981102627	10.61851661
80s	POC2	51	80	female	48.522813	29.762012	30.795692	21.435066	18.825856	1159.4571	0	3.106749391	0.043974	5.547166276	38.95733203
	POC2	60	87	female	51.906286	57.154144	54.604924	45.398328	50.922158	629.43822	43.5	4.01541499	0.0617157	7.614230683	46.70132948
	POC3	084	83	female	29.620106	44.098244	44.043986	46.193182	38.661993	1378.4956	10.5	7.847598483	0.1173859	7.963407124	15.60021617
	POC3	086	84	female	38.525901	33.8176	11.599335	30.420639	21.317433	636.50491	44.5	7.457504831	0.1165301	6.099127302	45.1040526
	POC3	088	82	female	43.059607	16.775045	18.222177	32.196805	17.999812	620.25692	36	5.160108102	0.0854356	7.12312889	27.87199255
	POC3	071	82	male	28.042703	37.486744	18.431258	23.059202	34.715335	446.95596	48	10.25695522	0.0917135	9.750668969	32.90337524
	POC3	058	83	male	32.630232	24.429518	19.315273	15.300156	23.207112	1387.2841	0	8.006438927	0.1172927	10.71129244	22.04999598
	POC3	078	88	male	22.242282	37.718333	13.438066	38.047011	41.550089	1166.223	3	9.66071378	0.1021605	9.798535644	26.84140875
	POC2	27	80	male	16.304923	16.614755	14.026827	15.762657	39.434656	1569.6213	0.5	15.85255236	0.0074363	13.98749854	62.79577623
	POC2	50	80	male	24.477648	24.463857	29.409643	18.052581	16.488665	726.54505	35.5	6.117143881	0.1027804	4.492758434	20.83159974
	POC2	53	80	male	37.10598	18.452174	12.688342	45.721293	36.496644	1435.3042	0	3.73851997	0.0560827	5.913470731	17.02612334
	POC2	56	80	male								11.79700007	0.0601963	13.60570008	77.74341279
	POC2	48	81	male	40.14983	17.242455	21.053384	18.089925	27.765132	461.44756	40.5	11.01642856	0.0764539	12.72661862	65.7136862
	POC2	49	81	male	51.801564	53.579231	34.139649	33.666672	28.600095	934.63038	24.5	1.395033119	0.0191239	3.218592715	16.74462151
	POC2	58	81	male	48.895266	24.784878	12.220502	18.367636	11.655276	252.22897	51	6.163164079	0.103491	5.623575636	30.77731264
	POC2	65	81	male	37.020139	49.797385	26.951071	49.782989	47.546983	903.11426	9	8.112177221	0.1170887	10.49120752	75.89743461
	POC2	66	82	male	38.204337	36.483556	26.265907	22.279609	39.870989	742.41793	33	8.77465086	0.1132951	8.922034824	30.70007018
	POC2	67	82	male	25.705692	48.203635	53.403169	57.077429	53.448557	1007.6187	15.5	12.90836242	0.0391027	14.42963955	77.79839669
	POC2	52	83	male	46.905917	16.144647	24.294574	13.771242	21.778051	397.5305	47.5	7.345590435	0.1160032	7.404243969	32.60819967
	POC3	091	82	male	41.913926	15.11865	16.094738	11.311534	36.407665	712.33576	48.5	11.66653667	0.0628808	12.0293755	46.79891614

	POC3	082	83	male	29.353862	33.029067	36.953059	13.31626	17.471341	930.97246	23	6.940532535	0.1130863	7.04865835	18.8537343
	POC3	085	80	male	38.386719	47.261817	44.191393	26.624636	42.791064	1056.4362	6.5	5.803099871	0.0975813	7.538957733	17.87079065
	POC3	090	81	male	39.579272	24.575609	27.308311	23.551683	28.214866	688.13919	44	7.849540728	0.1173864	7.772693906	27.53017595

List of raw data in figures 3D.

Figure 3D left:

sex	c-Fos-positive cells in EC / mm^2 with vehicle-injection	c-Fos-positive cells in EC / mm^2 with CNO-injection
male	156.32002	73.23325
	92.15475	56.33272
	188.16293	49.179558
	124.25139	34.262333
	78.85411	26.735142
	207.25134	71.565285
	121.30048	90.850586

Figure 3D right:

sex	Angular error (degrees) with vehicle-injection	Angular error (degrees) with CNO-injection
male	23.872286	84.50316
	40.171913	66.58912
	50.6057	129.04918
	15.001004	49.470203
	27.139854	104.34103
	73.67886	102.9987

List of raw data in figures 4A-4E and supplemental figure 4.

Figure 4A:

sex	♀	♀	♂	♀	♀	♀	♀	♀	♂	♂	♂	♀	♂	♂	♀	♂	♀	♀
Age (months)	4.6	4.6	4.89	4.89	4.6	4.6	3.75	3.75	3.91	3.29	4.6	4.6	4.89	4.6	3.26	2.99	2.89	2.89
genotype (PS19: +)											+	+	+	+	+	+	+	+
Angular error (degrees)	21.892876	46.907486	18.855839	20.498142	70.964944	15.126624	39.331017	42.445667	7.1196776	11.740884	17.481319	20.31645	48.942309	15.803096	30.378081	21.358879	67.401728	36.971243
sex	♂	♂	♂	♀	♀	♀	♂	♂	♂	♀	♂	♂	♂	♀	♀	♂	♀	♂
Age (months)	7.07	7.07	6.64	6.64	6.64	7.17	7.6	7.6	6.91	7.66	7.04	7.04	7.04	7.07	7.1	6.64	6.28	6.91
genotype (PS19: +)											+	+	+	+	+	+	+	+
Angular error (degrees)	36.7519	12.269181	24.688794	90.053189	38.241365	13.369051	16.552445	33.0613	48.423156	29.29444	26.112767	47.830078	19.669312	35.968363	117.76692	29.478702	86.03217	16.208952

Figure 4B and Supplemental figure 4:

sex	♀	♀	♂	♂	♀	♀	♀	♀	♂	♂	♀	♀	♀	♀	♂	♀	♀	♂
Age (months)	3.55	4.27	4.74	4.74	3.52	3.88	3.88	3.88	3.68	4.24	3.55	3.55	3.91	3.91	4.24	4.28	4.28	3.91
genotype (PS19: +)											+	+	+	+	+	+	+	+
Angular error (degrees)	42.654501	37.690697	20.51974	21.601828	27.044454	29.847623	30.62833	73.350965	35.106877	24.407512	11.333757	31.087757	35.320261	49.958985	26.973535	68.947984	13.750822	21.044408
AT8-positive cell bodies	0	0	0	0	0	0	0	0	0	0	0	0	0	0	2	0	0	0
sex	♀	♂	♂	♂	♀	♀	♂	♂	♂	♀	♀	♀	♂	♀	♂	♂	♂	♂
Age (months)	7.17	7.17	7.14	6.51	7.82	7.82	6.91	6.91	7.37	7.43	7.17	7.17	6.51	6.51	6.88	6.55	6.55	6.55
genotype (PS19: +)											+	+	+	+	+	+	+	+
Angular error (degrees)	39.329469	71.1032	17.852259	41.752644	34.059294	29.63239	22.497031	27.523919	27.402995	42.117536	30.760591	111.24597	34.272246	30.261736	75.972547	99.513527	50.476936	77.075686
AT8-positive cell bodies	0	0	ND	0	0	0	0	0	0	0	1	5	8	7	7	1.5	9	9

ND; not detected

Figure 4C, D and E:

sex		♀	♀	♀	♀	♀	♂	♂	♂	♀	♂	♀	♀	♀	♀	♂	♂	♂	♀
Age (months)		5.06	5.06	6.94	6.94	6.94	7	7	7	6.94	6.01	5.92	6.45	6.45	6.45	7	7	7	6.94
genotype (PS19: +)										+	+	+	+	+	+	+	+	+	+
Averaged total distance traveled (cm)	Day 1	248.5	647.1	605.7	147.73333	160.46667	403.7	399.43333	227.26667	436.63333	440.23333	472.8	296.56667	192.2	537.13333	189.86667	239.23333	450.46667	514.3
	Day 2	143.63333	502.7	751.2	356.66667	516.9	390.83333	378.06667	258.63333	609.83333	403.13333	311.13333	474.23333	847.23333	377.8	309.03333	310.8	143.46667	185.9
	Day 3	117.56667	209.26667	108.63333	213.5	210.2	307.3	137.3	144.7	254.66667	329.7	274.8	309.76667	557.66667	282.23333	211.43333	198.36667	610.23333	136.5
error score (x 10^4)		6.93048	8.13473	7.20698	7.53665	10.41977	6.23882	9.16449	7.90462	8.14345	10.07013	6.965	11.87884	8.11706	7.5135	6.93478	9.91441	8.55912	8.33026
Time spent in the T.Q. (%)		50.167598	34.692737	47.318436	38.268156	23.128492	56.871508	30.893855	37.430168	36.201117	23.184358	52.960894	13.575419	35.195531	42.849162	52.569832	25.698324	37.541899	37.877095

List of raw data in figures 5H-K.

Age (months)	Mouse ID.	Sex	Entorhinal cortex-left	Entorhinal cortex-right	CA3-left-anterior	CA3-right-anterior	CA1-left-anterior	CA1-right-anterior	DG-left-anterior	DG-right-anterior	CA3-left-posterior	CA3-right-posterior	CA1-left-posterior	CA1-right-posterior	DG-left-posterior	DG-right-posterior
4.0	407	female	0	0	0	0	0	0	0	0	0	0	0	0	0	1
			0	0					0	0	0	0	0	0		0
			0	0												
4.0	410	male	1	1	0	0	0	0	0	0	0	0	0	0	0	0
			0	2			0	0						0		
			0	1												
7.2	376	female	11	1	0	0	0	0	0	0			7		0	2
			8	2			0	0							0	1
			14	1												7
				2												3
6.3	381	male	3		11	1	1	1	0	0	0	1	1	1	1	7
			4				0	0	0	0	0	0	0	1	13	20
			0									0			3	25
			1									1			2	
6.3	384	female		21	2		0				0	0	2	0	0	0
				15	1		0				0	0	1	0	0	
				3									0		1	
6.1	391	female														
			8	10	0	0	0	1	4	0	0	0	0	1	25	0
			7	24	0	0	0	0	0		0	0	1	1	24	3
			1	3										0	22	1
9.0	PS31	male		21								16		16		40
				20								12		15		35
				3								12		14		28
				11												
				11												

				5												
9.0	PS32	male		12		17								7		25
				17		13								2		22
				15										4		27
9.0	PS33	male		14		20								29		21
				7		17								15		21
				13										11		37
9.0	PS35	male		7								4		1		31
				3								0		1		30
												0		2		35
														1		

List of raw data in supplemental figure 3.

sex	Angular error (degrees) with vehicle-injection	Angular error (degrees) with CNO-injection
male	11.023812	31.52404
	33.428032	36.059097
	15.375898	39.432217
	46.64464	54.55065
	48.94828	15.310135
	46.45676	30.344065

Supplemental references.

1. Braak H, Braak E. Frequency of stages of Alzheimer-related lesions in different age categories. *Neurobiol Aging*. Jul-Aug 1997;18(4):351-7. doi:10.1016/s0197-4580(97)00056-0
2. Jicha GA, Bowser R, Kazam IG, Davies P. Alz-50 and MC-1, a new monoclonal antibody raised to paired helical filaments, recognize conformational epitopes on recombinant tau. *J Neurosci Res*. Apr 15 1997;48(2):128-32. doi:10.1002/(sici)1097-4547(19970415)48:2<128::aid-jnr5>3.0.co;2-e
3. Gil M, Ancau M, Schlesiger MI, *et al*. Impaired path integration in mice with disrupted grid cell firing. *Nat Neurosci*. Jan 2018;21(1):81-91. doi:10.1038/s41593-017-0039-3
4. Jacob P-Y, Capitano F, Poucet B, Save E, Sargolini F. Path integration maintains spatial periodicity of grid cell firing in a 1D circular track. *Nature Communications*. 2019/02/19 2019;10(1):840. doi:10.1038/s41467-019-08795-w
